# Adult-onset leukodystrophies: a practical guide, recent treatment updates, and future directions

**DOI:** 10.3389/fneur.2023.1219324

**Published:** 2023-07-26

**Authors:** Karthik Muthusamy, Ajith Sivadasan, Luke Dixon, Sniya Sudhakar, Maya Thomas, Sumita Danda, Zbigniew K. Wszolek, Klaas Wierenga, Radhika Dhamija, Ralitza Gavrilova

**Affiliations:** ^1^Department of Clinical Genomics, Mayo Clinic, Jacksonville, FL, United States; ^2^Department of Neurological Sciences, Christian Medical College, Tamil Nadu, Vellore, India; ^3^Department of Radiology, Imperial College, NHS Trust, London, United Kingdom; ^4^Department of Radiology, Great Ormond Street Hospital, London, United Kingdom; ^5^Department of Medical Genetics, Christian Medical College, Vellore, Tamil Nadu, India; ^6^Department of Neurology, Mayo Clinic, Jacksonville, FL, United States; ^7^Department of Clinical Genomics and Neurology, Mayo Clinic, Phoenix, AZ, United States; ^8^Department of Clinical Genomics and Neurology, Mayo Clinic, Rochester, MN, United States

**Keywords:** adult-onset leukodystrophy, leukodystrophy, leukoencephalopathy, neurogenetics, neurometabolic disorder

## Abstract

Adult-onset leukodystrophies though individually rare are not uncommon. This group includes several disorders with isolated adult presentations, as well as several childhood leukodystrophies with attenuated phenotypes that present at a later age. Misdiagnoses often occur due to the clinical and radiological overlap with common acquired disorders such as infectious, immune, inflammatory, vascular, metabolic, and toxic etiologies. Increased prevalence of non-specific white matter changes in adult population poses challenges during diagnostic considerations. Clinico-radiological spectrum and molecular landscape of adult-onset leukodystrophies have not been completely elucidated at this time. Diagnostic approach is less well-standardized when compared to the childhood counterpart. Absence of family history and reduced penetrance in certain disorders frequently create a dilemma. Comprehensive evaluation and molecular confirmation when available helps in prognostication, early initiation of treatment in certain disorders, enrollment in clinical trials, and provides valuable information for the family for reproductive counseling. In this review article, we aimed to formulate an approach to adult-onset leukodystrophies that will be useful in routine practice, discuss common adult-onset leukodystrophies with usual and unusual presentations, neuroimaging findings, recent advances in treatment, acquired mimics, and provide an algorithm for comprehensive clinical, radiological, and genetic evaluation that will facilitate early diagnosis and consider active treatment options when available. A high index of suspicion, awareness of the clinico-radiological presentations, and comprehensive genetic evaluation are paramount because treatment options are available for several disorders when diagnosed early in the disease course.

## Introduction

Leukodystrophies are clinically and genetically heterogeneous disorders that are characterized by the common occurrence of white matter changes in the brain. A tremendous increase in our knowledge of pathophysiology, genomic and biomarker discoveries, and advanced MRI techniques that happened over the past decade has enabled us with promising translational research. Classification and practice of adult-onset leukodystrophies are often extrapolated from the pediatric literature, where it is well-standardized. Though there is a continuum with certain leukodystrophies occurring from childhood to adulthood with milder phenotypes, certain leukodystrophies present exclusively in the adult population. Several acquired disorders in this age group such as infections, immune-mediated, and toxin exposures can mimic the clinico-radiological presentation. Hence, ruling out acquired disorders remains the cornerstone during the initial evaluation. A three-generation family history, chronology of the neurologic symptoms and signs, search for relevant systemic associations, and pattern recognition in neuroimaging often provide valuable insights into the underlying etiology. Early diagnosis is essential as several of them are potentially treatable when diagnosed early in the disease course. Molecular confirmation is also valuable in screening at-risk family members in the pre-symptomatic state.

## Definitions

Leukodystrophies are defined as all genetic-derived diseases that primarily affect the white matter of the central nervous system, encompassing both the construction and maintenance of myelin as well as the key neuroglial cells involved (1). Leukoencephalopathies, on the other hand, are of higher taxonomic rank that covers both leukodystrophies and secondary acquired diseases that primarily affects white matter (1). Genetic leukoencephalopathies (gLE) are another term for genetic disorders with significant, if not sole, white matter involvement and/or diseases with pre-eminent systemic manifestations that eclipse the white matter pathology (2).

## Classification

The classification of leukodystrophies continues to evolve due to the growing understanding of the pathological underpinnings and the ongoing discovery of new genetic causes. The first report of a familial white matter disorder was described in 1899 by Pelizaeus (1). Over the last century, following this and further early pathological series, an initial myelin-centric classification of leukodystrophies was established (1). Over the last two decades, with the advent of whole-genome sequencing (WGS), whole-exome sequencing (WES), and modern imaging, there has been a paradigm shift that has greatly broadened the classes of leukodystrophies. In parallel with these advances, there has been a more precise definition of adult leukodystrophies which previously had been less recognized than the more readily encountered childhood-onset leukodystrophies. Presently, the classification of adult leukodystrophies mirrors childhood categorization, although the distribution and prevalence of subtypes within these categories diverge somewhat. Leukodystrophies can be pathologically categorized into myelin disorders, astrocytopathies, microgliopathies, leuko-axonopathies, genetic leuko-vasculopathies and inherited leukodystrophies of unknown origin (2, 3). Myelin disorders can be further subdivided into hypomyelinating leukoencephalopathies, demyelinating leukoencephalopathies, and leukoencephalopathies with myelin vacuolization (3). This classification, while useful as a framework, from a pragmatic point of view, does not necessarily account for the underlying molecular and metabolic causes, which has treatment implications, or the clinical presentation, which has diagnostic implications from a top-down perspective (1). To account for this, an alternative, complementary clinical framework has since been proposed that categorizes leukodystrophies by their pathogenetic process based on organelle dysfunction or impairment of selected metabolic pathways, and includes peroxisomal disorders, lysosomal disorders, mitochondrial respiratory chain disorders, defects in amino acid and organic acid metabolism, DNA repair disorders, genetic vasculopathies, translation defects, and defects in ion and water homoeostasis (2, 3). A simplified table on the classification of adult-onset leukodystrophies based on pathology is shown in Table 1. Interested readers are directed to review the tables in the listed references for further details including classification based on pathogenetic processes (2, 3). It is important to acknowledge that both frameworks have an inherent reductionism of complex pathophysiological processes, and that some of the leukodystrophies can straddle several classes.

**Table 1:** Classification of adult-onset leukodystrophies based on underlying pathology.

**Hypomyelination**
Pelizaeus-Merzbacher disease
POLR3-related disorders
**Demyelination**
Metachromatic leukodystrophy
Globoid cell leukodystrophy (Krabbe disease)
X-linked adrenoleukodystrophy (cerebral form)
Adrenomyeloneuropathy
*LMNB1*-related autosomal dominant demyelinating leukodystrophy
Cerebrotendinous xanthomatosis
**Myelin vacuolization**
X-linked Charcot-Marie-tooth disease
Phenylketonuria
Mitochondrial diseases with leukoencephalopathy
**Microgliopathies**
*CSF1R*-related leukoencephalopathy (adult-onset leukoencephalopathy with axonal
spheroids and pigmented glia)
**Astrocytopathies**
Alexander disease
*CLCN2*-related leukoencephalopathy
Vanishing white matter disease
**Leuko-axonopathies**
Gordon Holmes syndrome
Neuronal intranuclear inclusion disease
Leukoencephalopathy with brain stem and spinal cord involvement and lactate
elevation
*AARS2*-related leukoencephalopathy
**Leuko-vasculopathies**
Cerebral autosomal dominant arteriopathy with subcortical infarcts and
leukoencephalopathy
Cerebral autosomal recessive arteriopathy with subcortical infarcts and
leukoencephalopathy
Cathepsin A-related arteriopathy with strokes and leukoencephalopathy
*COL4A1*-related cerebral small vessel disease
Leukoencephalopathy with cysts and calcifications
Retinal vasculopathy with cerebral leukodystrophy and systemic manifestations
Fabry disease
**Other**
α-methyl-acyl-CoA-racemase (AMACR) deficiency
Fragile X-associated tremor ataxia syndrome

## Epidemiology

The genetic spectrum of childhood leukodystrophies has been well explored, with metachromatic leukodystrophy (MLD) widely regarded as common, accounting for up to 25.3% of childhood leukodystrophies (4). In contrast, the spectrum of adult leukodystrophies is much less well understood. This is partly related to their relative rarity, varied presentations, and perceived diagnostic challenge due to the greater proportion of confounding mimics such as multiple sclerosis and small vessel disease. The epidemiology of adult leukodystrophies also varies across studies due to different populations and inclusion criteria. In a 2015 European cohort study of 154 patients, cerebral autosomal-dominant arteriopathy with subcortical infarcts and leukoencephalopathy (CADASIL) was the most common diagnosis (33%), followed by vanishing white matter disease (VWMD), X-linked adrenoleukodystrophy (X-ALD), and *COL4A1*-related disorders (5). Since then, with the ever-increasing recognition of novel genes and further large-scale studies, the spectrum of adult leukodystrophies has continued to evolve. In a subsequent 2019 Japanese cohort, the newly characterized neuronal intranuclear inclusion disease (NIID) was found to surpass CADASIL as the most common cause of adult leukodystrophy (6). In a recent 2023 study of 309 adult Chinese patients with suspected genetic leukoencephalopathy, 201 patients were genetically diagnosed, while 108 patients remained undiagnosed (7). Of the genetically diagnosed, the most common category was leukovasculopathies (35%), followed by leuko-axonopathies (31%), myelin disorders (21%), microgliopathies (7%), and astrocytopathies (6%). Among the genetic leukovasculopathies, *NOTCH3* gene was the commonest, followed by *HTRA1* and *COL4A1/2* (7). The greatest variety of genetic causes was in the leuko-axonopathies group, of which *NOTCH2NLC* (NIID) was the most prevalent. Among the demyelinating leukoencephalopathies, adrenoleukodystrophy and Krabbe disease were the most common myelin disorders, while hypomyelinating leukoencephalopathies were rare (7). Among microgliopathy, *CSF1R*-related leukoencephalopathy was the only one detected. In the astrocytopathy group, leukoencephalopathy with vanishing white matter disease due to pathogenic variants in *EIF2B2-5* was the most frequent (7). While the proportion of undiagnosed genetic causes remains high in adults, with greater awareness and access to genetic testing, the number of unidentified cases continues to shrink.

## Clinical presentations: difference from childhood leukodystrophies

Leukodystrophies can present variably and at any age. In general, adult-onset leukodystrophies have a milder, sometimes stuttering course and a wider phenotypic spectrum with more neuropsychiatric and behavioral symptoms. The clinical presentation of a specific genetic mutation can also vary greatly; for instance, X-linked adrenoleukodystrophy (X-ALD) can be present in both childhood and adulthood in the same family (8). Typically, the age of onset inversely correlates with the rate of progression and severity (3, 9). Predominant symptoms in adult-onset leukodystrophies include motor impairment, neuropsychiatric symptoms, ataxia, and cognitive deficits. In contrast to children, psychiatric symptoms and/or slowly worsening cognitive deterioration can often be the first manifestation and may precede neurological signs years in advance (10). Psychiatric manifestations in adult-onset leukodystrophies can be separated into two groups: those with subtle, non-specific psychiatric symptoms (such as intellectual disability, disturbances in attention, and hyperactive behavior) that can be appreciated during childhood development (e.g., metachromatic leukodystrophy, adult-onset Alexander's disease, X-linked adrenoleukodystrophy, and *POLR3*-related leukodystrophy), and those which present solely in adulthood, often in the setting of worsening cognitive impairment in disorders such as CADASIL, *AARS2*-related leukoencephalopathy, and *CSF1R*-related leukoencephalopathy) (10).

Longstanding neuropsychiatric symptoms are sometimes only appreciated retrospectively following the onset of other neurological symptoms. Motor dysfunction is a prominent early neurological symptom in many adult-onset leukodystrophies, with progressive spastic paraparesis being the most recognized (11). Gait disturbances with gait ataxia (e.g., metachromatic leukodystrophy) or sensory long-tract signs (e.g., leukoencephalopathy with brainstem and spinal cord involvement and lactate elevation) are also common, albeit non-specific, feature (11). Extrapyramidal symptoms are less frequent but, if present, can serve as useful diagnostic clues, such as parkinsonism in *CSF1R*-related leukoencephalopathy, chorea in Gordon Holmes syndrome, and dystonia in *AARS2*-related leukoencephalopathy (11, 12). Bulbar or pseudobulbar palsies can be a distinctive early feature of adult-onset Alexander disease but, in the later stages, is non-specific and can occur in many adult-onset leukodystrophies (12). Lower limb sensory impairment and autonomic dysfunction (such as bladder and bowel incontinence) can also occur (e.g., adrenomyeloneuropathy and *LMNB1* autosomal-dominant leukodystrophy) (12). Peripheral neuropathy is recognized in certain leukodystrophies, such as metachromatic leukodystrophy and neuronal intranuclear inclusion disease (12, 13).

Non-neurological symptoms can provide useful clues to a specific diagnosis. Endocrine abnormalities are particularly important to detect, such as Addison disease (X-ALD), ovarian failure (*AARS-*related leukoencephalopathy, vanishing white matter disease), and hypogonadotropic hypogonadism (Gordon Holmes, *POLR3*-related disorders). Few other specific abnormalities can also point to particular leukodystrophies, e.g., tendon xanthomata and cataracts in cerebrotendinous xanthomatosis, and dental abnormalities in *POLR3*-related disorders (11, 12).

A comprehensive list of the neurologic and systemic clues is shown in Tables 2, 3.

**Table 2:** Neurologic clues in the diagnosis of adult-onset leukodystrophies.

**Prominent and early neurologic symptom**	**Disorders**
Cognitive decline	FXTAS, NIID, *CSF1R*-related leukoencephalopathy, CTX, CADASIL, CARASIL, *AARS2*-related leukoencephalopathy
Psychiatric manifestations	MLD, *CSF1R*-related leukoencephalopathy, X-ALD, FXTAS, CTX, *AARS2*-related leukoencephalopathy
Seizures	*CSF1R*-related leukoencephalopathy, CTX, LCC, VWMD, LBSL AMACRD, MELAS
Migraines	Mitochondrial disorders, AMACRD, CADASIL, *COL4A1*-related CSVD.
Strokes/Transient ischemic attacks	CADASIL, CARASIL, *COL4A1*-related CSVD, Homocystinuria, Fabry disease
Stroke-like episodes	MELAS, AMACRD, NIID
Encephalopathy	MELAS, NIID, VWMD, AMACRD
**Movement disorders:** Chorea Dystonia Parkinsonism Tremors	Gordon Holmes syndrome CTX, *AARS2*-related leukoencephalopathy *CSF1R*-related leukoencephalopathy, NIID, FXTAS, CTX, *AARS2*-related leukoencephalopathy ADLD, NIID, FXTAS, *AARS2*-related leukoencephalopathy
**Spasticity:** Hemiparesis Quadriparesis Paraparesis	Krabbe disease Krabbe disease, AMN, hypomyelinating leukodystrophies AMN, APBD, hypomyelinating leukodystrophies
Cerebellar ataxia	CTX, ADLD, Gordon Holmes, NIID, hypomyelinating leukodystrophies, *CLCN2*-related leukoencephalopathy
**Brain stem signs** Extraocular movement abnormalities Palatal myoclonus Bulbar weakness	Alexander disease, FXTAS, MELAS, Alexander disease, CTX Alexander disease, APBD
**Vision** Optic atrophy Retinal dystrophy	X-ALD, late-onset biotinidase deficiency, MLD, Krabbe disease, PMD, mitochondrial disorders, *CLCN2*-related leukoencephalopathy Peroxisomal disorders, *CLCN2*-related leukoencephalopathy
Hearing impairment	Late-onset biotinidase deficiency, X-ALD
Autonomic disturbances Early bladder symptoms	ADLD, APBD, NIID, FXTAS APBD, AMN, ADLD
Neuropathy	CTX, APBD, NIID, FXTAS, MLD, ALD, AMN, LBSL, Krabbe disease, AMACRD
Myopathy	NIID, mitochondrial disorders, AMACRD
Myelopathy	AMN, spinal variant of CTX, LBSL, APBD
Increased intracranial pressure	LCC
**Clinical course** Prolonged periods of stable clinical course Rapid decline with trauma and intercurrent illnesses Rare relapsing-remitting course	Krabbe disease, hypomyelinating leukodystrophies VWMD, ADLD, VWMD, LBSL, X-ALD Alexander disease, APBD

ADLD, autosomal-dominant adult-onset demyelinating leukodystrophy; AMACRD, α-methyl-acyl-CoA-racemase deficiency; APBD, adult polyglucosan body disease; AMN, adrenomyeloneuropathy; CADASIL, cerebral autosomal-dominant arteriopathy with subcortical infarcts and leukoencephalopathy; CARASIL, cerebral autosomal recessive arteriopathy with subcortical infarcts and leukoencephalopathy; CTX, cerebrotendinous xanthomatosis; FXTAS, fragile X-associated tremor ataxia syndrome; LBSL, leukoencephalopathy with brain stem and spinal cord involvement and lactate elevation; LCC, leukoencephalopathy, calcifications, and cysts, MELAS, mitochondrial encephalomyopathy with lactic acidosis and stroke-like episodes; MLD, metachromatic leukodystrophy; NIID, neuronal intranuclear inclusion disease, VWMD, vanishing white matter disease; X-ALD, X-linked adrenoleukodystrophy.

**Table 3:** Systemic clues in the diagnosis of adult-onset leukodystrophies.

**Skin**	
Alopecia Skin rash Ichthyosis Pigmentation	CARASIL, Biotinidase deficiency, AMN Biotinidase deficiency Refsum disease, Sjogren-Larsson syndrome X-ALD, AMN
**Progeroid features**	**CRMCC, Cockayne syndrome**
**Eyes**	
Cataract	CTX, mitochondrial disorders, hypomyelinating leukodystrophies, *COL4A1*-related disorders
Axenfeld-Rieger anomaly	*COL4A1*-related disorders
Retinal arterial torturosity/exudates	*COL4A1*-related disorders, CRMCC
**Dental**	
Hypodontia, oligodontia	*POLR3*-related hypomyelinating leukodystrophy
**Cardiac involvement**	
Cardiomyopathy	APBD, Fabry disease, *AARS2*-related leukoencephalopathy
Conduction disturbances Premature atherosclerosis	Fabry disease CTX
**Endocrine**	
Hypogonadism	Gordon Holmes syndrome, *POLR3*-related hypomyelinating leukodystrophy, AMN, VWMD, *AARS2*-related leukodystrophy, FXTAS, *CLCN2*-related leukoencephalopathy
Premature ovarian insufficiency	Galactosemia, FXTAS, VWMD, *AARS2*-related leukoencephalopathy
Addison disease	X-ALD, AMN
Growth hormone deficiency	*POL3*-related hypomyelinating leukodystrophy
**Gastrointestinal involvement**	
Cholecystitis, gall bladder polyps	MLD
Dysmotility Malabsorption GI bleeding	MNGIE CTX CRMCC
**Liver involvement**	**AMACRD, CTX, APBD, CRMCC**
**Skeletal involvement**	
Bone cysts Osteoporosis	PLOSL CTX
**Tendon xanthomas**	**CTX**
**Respiratory insufficiency**	**CTX**
**Renal involvement**	***COL4A1*** **-related disorders**
**Spondylosis deformans**	**CARASIL**

AMACR deficiency, α-methyl-acyl-CoA-racemase deficiency; APBD, adult polyglucosan body disease; AMN, adrenomyeloneuropathy; CARASIL, cerebral autosomal recessive arteriopathy with subcortical infarcts and leukoencephalopathy; CRMCC, cerebro-retinal microangiopathy with calcifications and cysts; CTX, cerebrotendinous xanthomatosis; FXTAS, fragile X-associated tremor ataxia syndrome; MLD, metachromatic leukodystrophy; MNGIE, mitochondrial neuro gastro intestinal encephalomyopathy; PLOSL, Polycystic lipomembranous osteodysplasia with sclerosing leukoencephalopathy; VWMD, vanishing white matter disease; X-ALD, X linked adrenoleukodystrophy.

## Imaging presentations: difference from childhood leukodystrophies

Clinical features are frequently non-specific, and initially often subtle in adult-onset leukodystrophies. The diagnostic challenge in adults is further compounded by the many potential acquired causes. Magnetic resonance imaging (MRI), with its high sensitivity for abnormal white matter, is usually the pre-eminent diagnostic tool to suggest a white matter disease, and presently, MRI patterns in conjunction with clinical features are still used to classify and narrow the differential diagnosis provisionally (3, 11). In certain cases, with specific imaging, clinical and biochemistry findings, it may also be possible to reach a definitive diagnosis prior to genetic testing. Considering this, a classification based on imaging patterns is still pragmatically useful as an approximate surrogate for underlying pathology. Based on MRI features, leukodystrophies can be simplistically classified into two groups: hypomyelinating and demyelinating. The hypomyelinating pattern is a relatively discrete group of leukodystrophies which, on MRI, is characterized by mild T2W hyperintensity and near normal/normal T1W signal, whereas the demyelinating pattern covers a broader and more heterogenous group of leukodystrophies which on MRI is defined by marked T1W hypointensity and T2W hyperintensity. It is important to understand that these patterns do not entirely correlate with underlying pathology. Nevertheless, the hypomyelinating pattern is predominantly observed in pathologically proven hypomyelinating leukodystrophies such as PMD, while the demyelinating pattern generally correlates with pathologically proven demyelinating leukodystrophies such as MLD (14). In addition to categorizing these patterns, it is also useful to define the anatomical structures involved, for instance, the brainstem or basal ganglia, and the presence of specific imaging features, such as calcifications, cysts, microbleeds, and infarcts. The finding of persistent diffusion restriction on DWI, which remains for long periods of time, beyond the transient nature of ischemia and demyelination, is a particularly useful feature in suggesting certain adult leukodystrophies, and in the discrimination from other potentially acquired causes. This observation is thought in many cases to reflect intramyelinic edema, and is particularly recognized in *CSF1R*-related leukoencephalopathy, *AARS2*-related leukodystrophy, and neuronal intranuclear inclusion disease (NIID), among others (15). Other imaging findings that are traditionally purported to favor acquired adult white matter disease include steroid responsiveness, asymmetrical multifocal white matter abnormalities, enhancement, cervical spinal cord involvement, and the presence of mass-like lesions (11, 12). These findings remain useful, but with the broadening characterization of “atypical” phenotypes, it is important not to be dogmatic, for instance, X-ALD can present with asymmetrical abnormalities, and certain other genetic CNS disorders can mimic acquired neuroinflammatory disorders presenting with enhancing and mass-like lesions, for instance, X-ALD, MLD, retinal vasculopathy with cerebral leukoencephalopathy and systemic manifestations, and *CTLA-4* haploinsufficiency (5). A clinically useful algorithmic approach encompassing family history and radiological findings is provided later in the paper.

**Figure 1:**
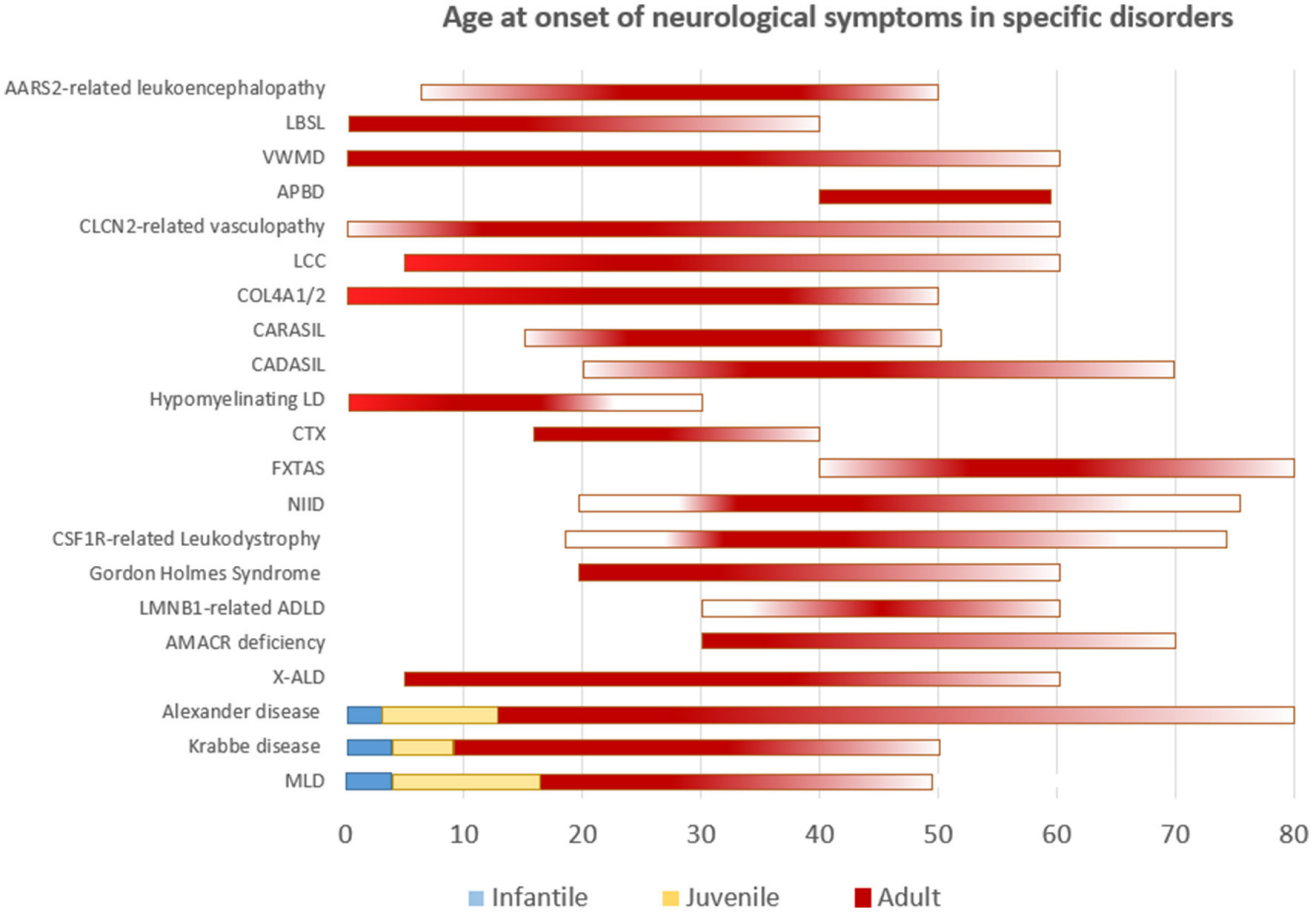
Simplified representation of the age of onset of neurological symptoms in different disorders. Disorders categorized according to well-defined age groups are depicted as such. Others are represented as a continuum with gradients portraying frequency.

Though the list of adult leukodystrophies is extensive, we intend to cover the common disorders that are encountered in clinical practice. A simplified representation of age at onset of neurological manifestations in specific leukodystrophies is shown in Figure 1. It should be understood that exception to the rule, and outliers exists for many of adult-onset leukodystrophies with regards to age at onset of symptoms.

### Metachromatic leukodystrophy

Metachromatic leukodystrophy (MIM# 250100) is a lysosomal disorder with autosomal recessive inheritance due to biallelic pathogenic variants in the *ARSA* gene (16). Rarely, deficiency of a sphingolipid activator protein (*PSAP*, MIM# 249900) that is required for the formation of the substrate–enzyme complex can result in similar clinical and radiological manifestations (16). The deficiency of the enzyme or the activator protein leads to the accumulation of sulfated glycosphingolipids in the cerebral white matter and peripheral nerves, resulting in demyelination and a progressive neurodegenerative course. Based on age at symptom onset, MLD has been classified as infantile, juvenile, and adult-onset forms. Juvenile MLD is characterized by the onset of symptoms from 3 to 16 years of age, and the occurrence of initial symptoms beyond age 16 is classified as adult MLD (16). The onset of symptoms as late as fourth or fifth decade is known. Adult-onset presentation is rare, and accounts for approximately 20% of all cases (16). Initial manifestations typically include intellectual and behavioral concerns such as personality changes, emotional lability, psychosis, and cognitive decline, followed by motor symptoms such as progressive incoordination, spasticity, seizures, and peripheral neuropathy. Misdiagnosis with schizophrenia, depression and psychosis are common in earlier stages of the disease (17). Rare presentation with isolated neuropathy is known (18). Since the sulfatides accumulate in the gallbladder, cholecystitis, gall balder polyps, and carcinoma are recognized complications (16).

MRI of the brain demonstrates symmetric confluent periventricular and deep white matter changes with a tigroid pattern that represents sparing of white matter in the perivenular regions. Corpus callosum is usually involved. White matter changes in adult onset are usually frontal predominant, and progresses later to involve parieto-occipital regions (19). Subcortical U fibers are spared in the initial stages. Variable hyperintensity is noted in DWI images. Progressive brain atrophy ensues in later stages (19). MRI scoring method has been established that helps in defining the natural history and monitoring disease response in treatment trials (20).

Low Aryl sulfatase enzyme levels can be seen with pseudodeficiencies, hence molecular confirmation is essential. Also, normal levels of the enzyme do not rule out MLD, as that could happen with activator protein deficiency. Urine sulfatides is a helpful screening test as it would be elevated in all cases of MLD regardless of the enzyme or activator protein deficiency, whereas it would be normal in pseudodeficiencies (16). Hematopoietic stem cell transplantation (HSCT) in earlier stages of adult MLD has been shown to be beneficial in arresting clinical progression (21). Gene therapy has been established for childhood-onset MLD.

### Krabbe disease

Globoid cell leukodystrophy or Krabbe disease is an autosomal recessive disorder caused by biallelic pathogenic variants in *GALC* (MIM# 245200) or *PSAP* (MIM# 611722) that leads to the deficiency of the enzyme galactocerebrosidase, or its activator protein saposin (22). This deficiency results in the accumulation of galactocerebroside and psychosine that eventually leads to oligodendrocyte apoptosis and gliosis, and results in clinical symptoms. Krabbe disease is categorized as early infantile, late infantile, juvenile, and adult-onset subtypes based on the age at the onset of symptoms. Clinical and radiological presentations are quite different for each subtype (22). While early onset variant have a relentlessly progressive course with loss of acquired developmental milestones along with spasticity, seizures, visual and mental deterioration, followed by decerebrate posturing and death, juvenile and adult-onset types manifest gradually progressive spastic hemiparesis or quadriparesis, along with varying combinations of visual impairment, ataxia, and neuropathy (23).

**Figure 2:**
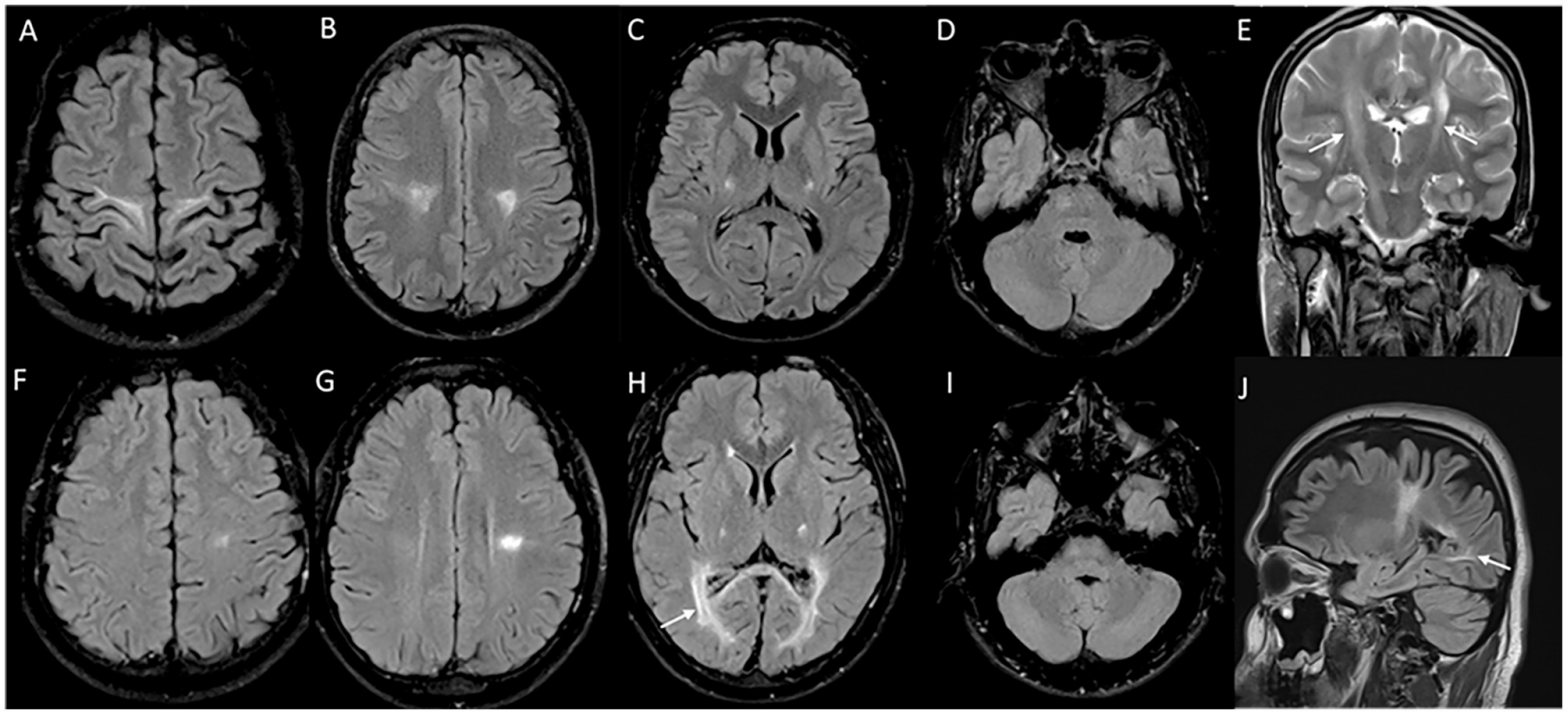
MRI brain spectrum in adult-onset Krabbe disease. T2 axial FLAIR sequences **(A–D)** and T2 coronal **(E)** of a 36-year-old male with the subacute onset and progressive spasticity due to Krabbe disease demonstrate symmetric signal changes in the motor cortex extending downward along the corticospinal tract in the perirolandic region, posterior limb of the internal capsule **(C)** and pons **(D)**, and garland-like appearance of the CST demonstrated in the coronal image [arrows in **(E)**]. Note the absence of splenial and adjacent parietooccipital white matter involvement **(E)**. T2 FLAIR axial **(F–I)** and T2 sagittal FLAIR **(J)** of another individual with late-onset Krabbe disease demonstrate similar findings along with additional splenial and parieto-occipital white matter involvement [arrows in **(H, J)**].

MRI brain in juvenile and adult-onset Krabbe disease show signal changes along the corticospinal tract from the motor cortex through its path in the corpus callosum, posterior limb of the internal capsule, cerebral peduncles, and brain stem. Signal changes can be asymmetric with unilateral corticospinal tract involvement in the earlier stages corresponding to the clinical deficit, and is usually followed much later by the involvement of the contralateral side and eventual quadriparesis (23). In juvenile onset and a proportion of adult-onset types, signal changes involving the posterior periventricular and deep white matter are also characteristically seen, and can mimic adrenoleukodystrophy (Figure 2). In adult-onset Krabbe disease, the corticospinal tract involvement can be seen isolated, or in combination with the posterior periventricular white matter involvement (22, 23). Periventricular and frontal white matter involvement can be seen in later stages of the disease. An imaging scoring system is available for the objective measurement of disease extent and severity that guides in tracking the radiological progression to assess the natural history and monitor therapy (24). Rare instances of normal MRI in the symptomatic phase are known (25).

Clinical course in juvenile and adult-onset Krabbe disease is highly variable with varying periods of clinical and imaging stability (23). Measurement of blood and CSF psychosine levels can aid in assessing the disease progression (26). Hematopoietic stem cell transplantation (HSCT) is considered for mildly symptomatic individuals with later onset disease with evidence of clinical progression (27). Given the heterogeneity in the clinical presentations and the disease course, candidates for HSCT need to be considered on a case-to-case basis (27).

### Alexander disease

Alexander disease (MIM# 203450) is an autosomal-dominant leukodystrophy due to heterozygous pathogenic variants in *GFAP* (28, 29). The disease mechanism is considered to be a gain of function mutation that disrupts the dimerization of intermediate filaments and leads to abnormal protein accumulation and collapse of the cytoskeleton (29). Clinical presentations are known to occur across all ages, and are categorized as neonatal, infantile, juvenile, and adult forms (30). While macrocephaly and regression of attained developmental milestones with rapid neurologic deterioration to death are the characteristic clinical course with neonatal and infantile-onset forms, late-onset forms have milder clinical severity and a slower neurologic decline (30). Juvenile forms have some clinical and imaging overlap with the adult form, and this spectrum probably represents a continuum (30).

**Figure 3:**
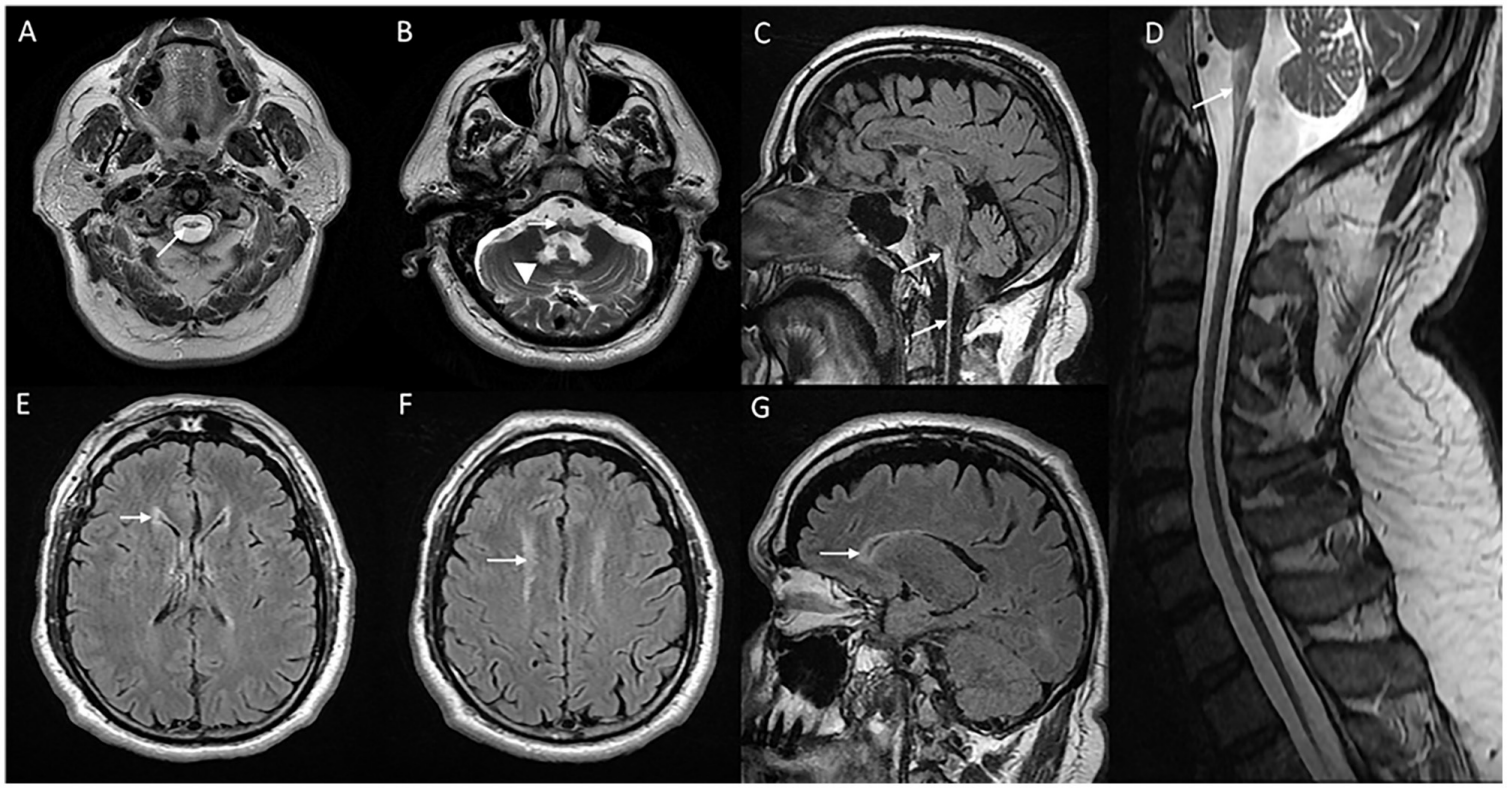
MRI of brain and cervical cord in adult-onset Alexander disease. T2W axial sections with marked atrophy and intrinsic signal changes in the upper cervical cord **(A)** and lower medulla **(B)** along with symmetric signal changes in the dentate hilum of the cerebellum [arrowhead in **(B)**]. T2W sagittal FLAIR imaging demonstrates signal changes and atrophy of the medulla and upper cervical cord with a “tad pole” appearance **(C)**. T2W sagittal spine imaging **(D)** reveals diffuse atrophy of the cervical cord. T2W FLAIR axial **(E, F)** and sagittal **(G)** demonstrate signal changes along the wall of lateral ventricles predominantly along the frontal horns.

The onset of symptoms can be as late as the sixth or seventh decade of life. Clinical manifestations are predominantly localized to the brain stem with bulbar, and pseudobulbar signs with symptoms such as dysphagia, vocal cord paralysis, dysphonia, dysarthria, and palatal myoclonus, followed by pyramidal and cerebellar signs (31). Sleep disturbances, extraocular movement abnormalities, and autonomic dysfunction are common. Presentations with hemiparesis, extrapyramidal symptoms, and relapsing-remitting clinical course have been described rarely (32). Cognitive deficits and epilepsy are uncommon. Refractile vomiting and failure to thrive are seen in individuals along the juvenile onset spectrum (30). MRI brain (Figure 3) classically demonstrate T2W signal changes in the anterior portion of the medulla and dentate hilum, along with the prominent atrophy of the medulla and upper cervical cord leading to “tad pole” appearance (33). Supratentorial white matter changes are rare and includes signal changes in the frontal horn of the lateral ventricles, cysts along the ventricular wall described as “garland appearance,” and mild to moderate signal changes in cerebral white matter (34). Transient swelling of the medulla before the eventual atrophy, and tumor-like presentations have been described in the literature (35, 36). Pathologic hallmark is the finding of rosenthal fibers in astrocytes on brain biopsy or autopsy (37). Non-specific clinical presentation in adult-onset cases, as well as the brain stem changes in imaging can cause diagnostic dilemma, and hence misdiagnosis with other neurodegenerative disorders, immune/inflammatory disorders, and rarely tumors are common (37).

While penetrance appears to be complete in early-onset forms, incomplete penetrance with several asymptomatic family members and variable expressivity are known with adult-onset forms (30). Though genotype–phenotype correlations are not always possible, recurrent pathogenic variants in *GFAP* are known to be associated with specific age at onset phenotypes (18, 30). Treatment is essentially supportive.

### Peroxisomal disorders

#### X-linked adrenoleukodystrophy

X-linked adrenoleukodystrophy (MIM # 300100) is the most common peroxisomal disorder encountered in clinical practice. X-ALD is caused by hemizygous pathogenic variants in the *ABCD1* gene. *ABCD1* gene encodes for the peroxisomal membrane protein that is involved in the transport of very long-chain fatty acids (VLCFA) (38). Pathogenic variants in *ABCD1* lead to the accumulation of VLCFA in various organs including the brain and adrenal glands (38). Significant intra and interfamilial clinical variability exist, and there is no clear phenotype–genotype correlation (38). Cerebral forms of X-ALD are sub-grouped as childhood, adolescent, and adult-onset types based on the age at the onset of symptoms. Clinical symptom onset from 11 to 21 years of age is classified as adolescent onset and onset anytime beyond the age of 21 is termed as adult-onset phenotype (38). Adult-onset cerebral X-ALD is rare and accounts for 3–5% of total cases (38). Initial presentation of adult cerebral X-ALD is usually one of non-specific psychiatric and behavioral symptoms such as personality changes, schizophrenia, and bipolar-like disorder, and these manifestations usually antedate the gait disturbances by several years (39, 40). Neurologic manifestations apart from the neuropsychiatric manifestations include progressive spasticity, cerebellar ataxia, cognitive decline, vision changes, seizures, bladder and bowel incontinence, and neuropathy (39). Clinical progression is slower when compared to childhood cerebral forms. Signs of adrenal insufficiency such as hyperpigmentation, hypotension, and electrolyte abnormalities are commonly seen. Asymmetric clinical involvement and slowly progressive pontocerebellar phenotypes are known in adult-onset forms, though rare (41–43).

**Figure 4:**
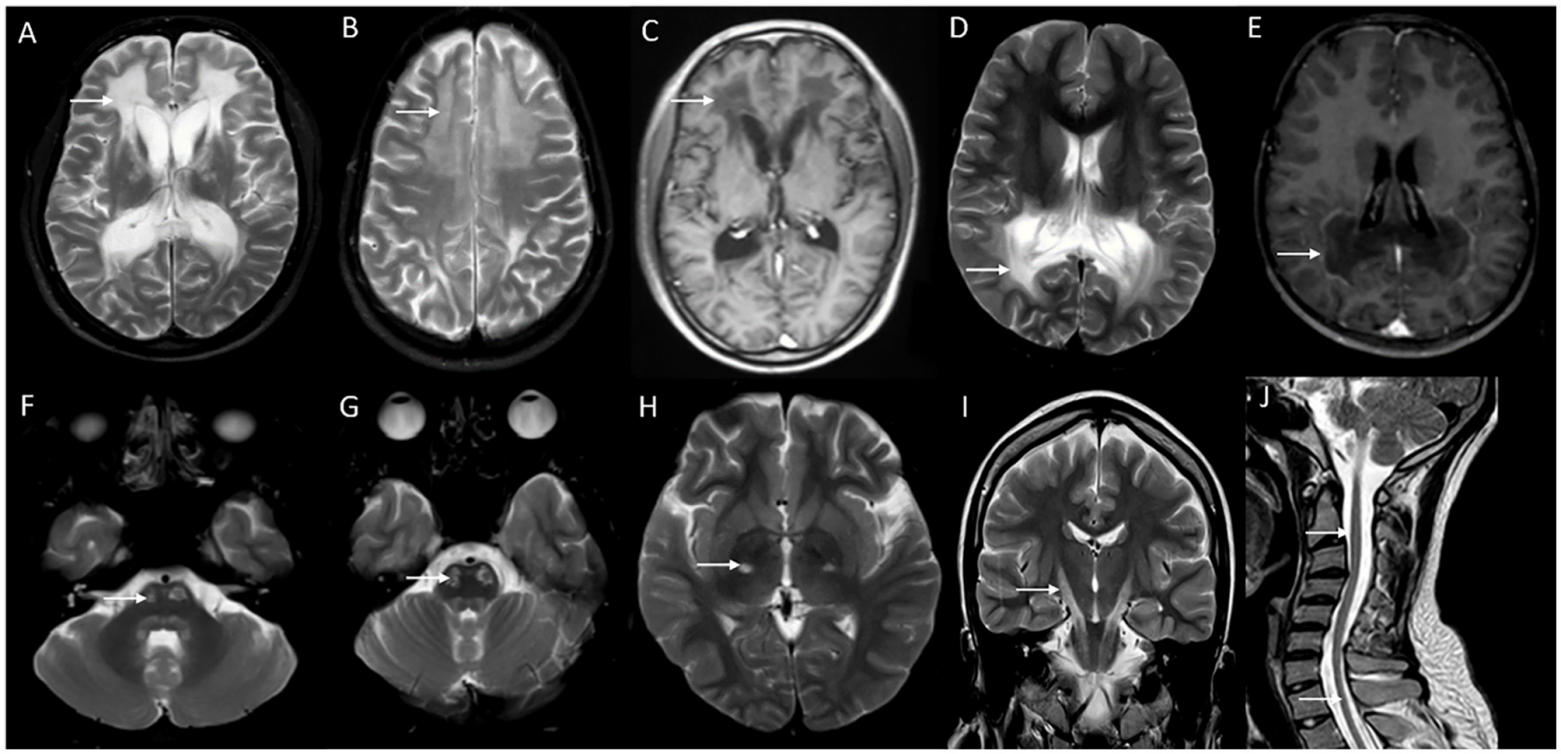
Neuroimaging spectrum in X-ALD **(A–E)** and AMN **(F–J)**. MRI brain T2 axial sequences **(A, B)** in the frontal/reverse variant of X-ALD reveal confluent subcortical, deep, and periventricular white matter hyperintensity involving frontal lobes in a symmetric distribution with associated volume loss. Note similar changes of less severe magnitude in the parieto-occipital regions and involvement of corpus callosum. T1 post-contrast image **(C)** shows subtle enhancement along the periphery of the involved frontal white matter. MRI T2 axial and T1 post-contrast (D and E) in classic X-ALD reveals confluent white matter hyperintensity involving the splenium of the corpus callosum and adjacent parieto-occipital white matter along with the contrast enhancement of the advancing margins. MRI T2W sequences in an individual with adrenomyeloneuropathy: axial **(F, G, H)** and coronal **(I)** sequences with signal changes along the corticospinal tract in the brain stem and posterior limb of the internal capsule. Note the signal changes stopping at the posterior limb of the internal capsule and not extending to the cortex. Superior extension of corticospinal tract signal changes beyond the posterior limb of the internal capsule represents the cerebral involvement of ALD; T2 sagittal MRI of the spine reveals marked diffuse cervical and thoracic cord thinning.

Neuroimaging findings are almost similar to childhood-onset forms with near symmetric confluent inflammatory demyelination of the splenium of the corpus callosum extending into the deep white matter of the parietal and occipital lobes with enhancement of the advancing border, along with the involvement of visual and auditory tracts (Figure 4). Involvement of splenium of the corpus callosum is usually the earliest imaging abnormality (38). Frontal predominant white matter involvement termed as frontal variant or reverse pattern of X-ALD (Figure 4), and isolated cerebellar white matter, dentate nuclei, and brain stem tracts involvement in imaging termed as the spinocerebellar ataxia or olivopontocerebellar atrophy variant of X-ALD are known and is most often seen with adult and adolescent-onset forms (43, 44). Asymmetric and tumor-like imaging presentations have been described as well (41, 45). The Loes score is the neuroimaging scoring of the areas of involvement that assess the MRI severity and is used for treatment decisions and follow-up (46).

Elevated levels of VLCFA with altered ratios of C26 and C24 to C22 are the pathognomonic lab finding, and is confirmed by molecular analysis of the *ABCD1* gene. HSCT is the treatment of choice for adolescents and adults with early stages of cerebral involvement (47).

**Adrenomyeloneuropathy (AMN)** manifests as slowly progressive spastic paraparesis in adult men in their third to fifth decades (48). Accompanying features are lower extremity weakness along with sensory disturbances, bladder and bowel incontinence, testicular insufficiency, exaggerated male pattern baldness, and neuropathy (38). Pathology is non-inflammatory axonal degeneration of the spinal tracts and peripheral nerves, and the symptoms usually progress gradually over decades (49). Nearly all males and ~60% of females with pathogenic variants in *ABCD1* develop symptoms of myeloneuropathy. Women usually present in their sixth decade or later and the progression is much slower than men with AMN (48). Approximately 70% of men with AMN have impaired adrenocortical function when neurologic symptoms are noted (48).

Approximately 40% of individuals with AMN have some degree of brain involvement in neuroimaging. In approximately 20% of individuals with AMN, brain involvement eventually progresses and leads to cognitive and behavioral decline implying cerebral involvement (50). Clinical manifestations of cerebral involvement in individuals with AMN are identical to those observed with primary adult cerebral ALD, with the additional symptom of pre-existing myelopathy. Current guidelines on surveillance in individuals with pathogenic variants in *ABCD1* is to perform MRI of the brain annually, as the development of cerebral changes even in late adulthood is known (50).

Neuroimaging of the brain in AMN is usually normal, or could show signal changes involving the pyramidal tracts in the internal capsules and brain stem (Figure 4). These abnormalities reflect the wallerian degeneration of long-standing myelopathy and do not implicate a cerebral involvement. Signal changes involving pyramidal tracts that extend upwards into the white matter of the centrum semiovale indicate cerebral involvement. MRI of the spinal cord shows non-specific atrophy without any signal changes (51). There is no specific treatment for AMN apart from the symptomatic management of spasticity and bladder functions. Timely identification and treatment of adrenal involvement are essential.

#### α-methyl-acyl-CoA-racemase deficiency (AMACR deficiency)

AMACR deficiency (MIM# 614307) is a rare autosomal recessive peroxisomal disorder with the deficiency of the single peroxisomal enzyme due to biallelic pathogenic variants in the *AMACR* gene. This leads to the accumulation of incorrect isomers of pristanic acid (52). Clinical manifestations are heterogeneous with variable age at the onset of symptoms ranging from infancy to adulthood as late as the seventh decade (52). While infants present with cholestasis and fat-soluble vitamin deficiencies, adult-onset presentations are essentially neurological and include recurrent encephalopathy, seizures, hemispheric symptoms with stroke-like episodes, migraine, cognitive decline, spasticity, tremors, pigmentary retinopathy, and sensorimotor axonal neuropathy (52–54). Psychiatric manifestations and muscle involvement with elevated creatine kinase levels are known (55). Pregnancy, fasting, and fever are the common precipitating factors for acute worsening. Varying periods of clinical stability occur, though most affected individuals eventually develop chronic progressive neurologic symptoms (52).

Symmetric signal changes involving thalami, mid-brain, pons, and cerebellar afferent and efferent tracts are the characteristic neuroimaging findings (56). In acute encephalopathy presentations with stroke-like episodes and seizures, neuroimaging reveals signal changes involving the corresponding cortex and deep white matter abnormalities with diffusion restriction (52, 56). Elevated level of pristanic acid is the diagnostic biochemical finding. Mildly elevated liver enzymes and accumulation of bile acid intermediates have been documented. Dietary exclusion of phytanic and pristanic acids has been shown to be beneficial (52).

### *LMNB1*-related autosomal-dominant leukodystrophy (autosomal-dominant adult-onset demyelinating leukodystrophy)

*LMNB1*-related autosomal-dominant leukodystrophy (MIM# 169500) is a rare exclusive adult-onset progressive leukodystrophy caused by mono-allelic pathogenic variants in *LMNB1* *(**57**)*. Symptoms usually start from the fourth or fifth decade with autonomic symptoms including bladder and bowel dysfunction, orthostatic hypotension, temperature dysregulation, and sweating abnormalities along with tremors, cerebellar ataxia, and spasticity with gait dysfunction predominantly involving lower limbs (57). Cognitive decline and pseudobulbar symptoms typically manifest late in the disease course. Sensorineural hearing loss and worsening of gait with intercurrent illnesses have been described rarely (57).

**Figure 5:**
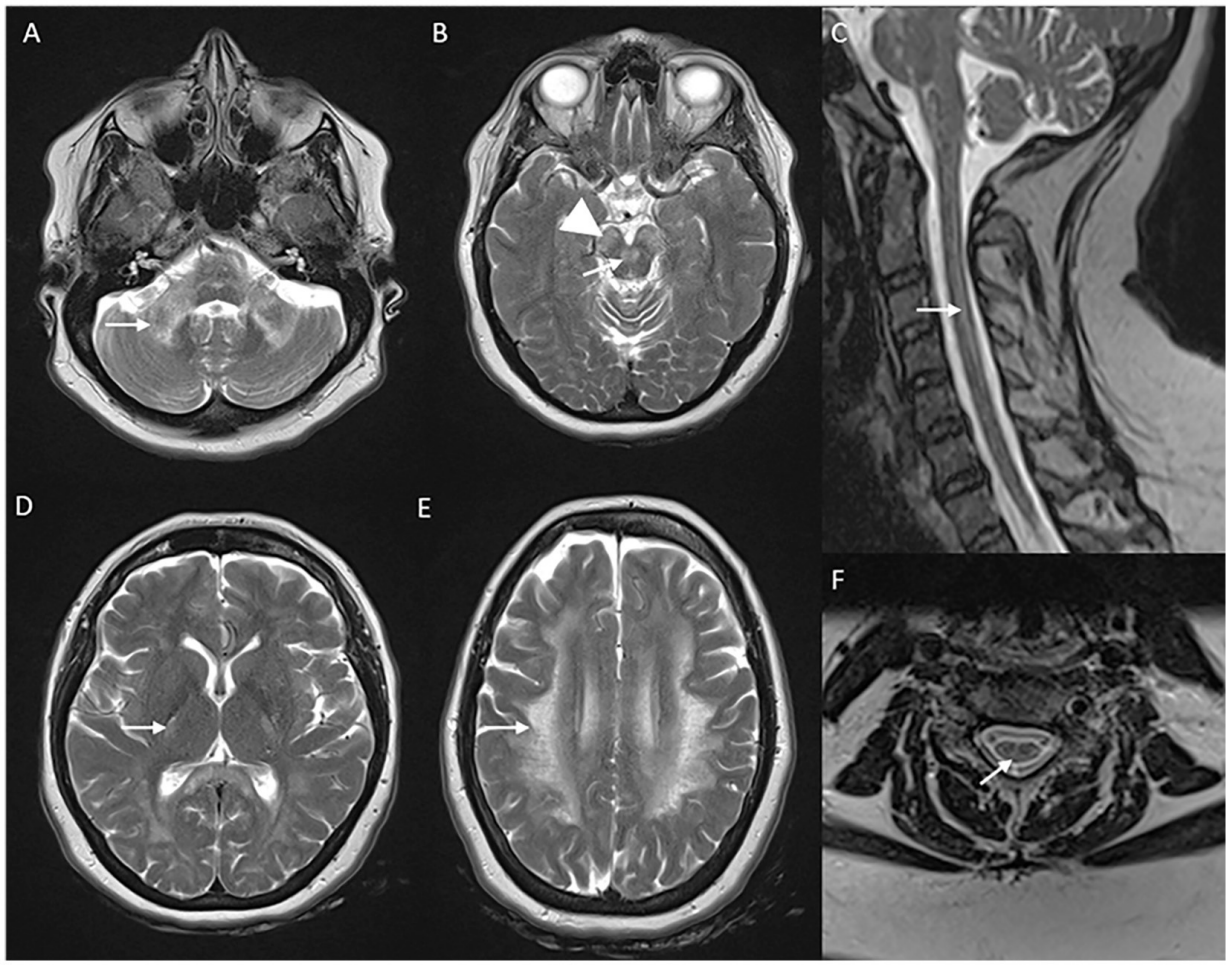
Neuroimaging in *LMNB1*-related autosomal-dominant leukodystrophy (autosomal-dominant adult-onset demyelinating leukodystrophy). MRI brain T2W axial section revealing symmetric signal changes in middle cerebellar peduncles **(A)**, medial longitudinal fasciculus (arrow in B), crus cerebri [arrowhead in **(B)**], posterior limb of the internal capsule **(D)**, and confluent, extensive subcortical and deep white matter changes in high frontoparietal regions. T2W sagittal **(C)** and axial **(F)** spine images show long segment signal changes along the cervical cord with the involvement of the lateral and dorsal columns.

Classic MRI brain findings include signal changes along the corticospinal tract and deep white matter changes involving frontal, parietal, and occipital lobes (57). Subcortical U fibers and periventricular rim of white matter are usually spared. The temporal lobe is usually involved later in the disease course. Symmetric T2W signal changes involving the brain stem with medial lemniscus, decussation of superior cerebellar peduncles, and middle cerebellar peduncle are typical findings (58). A trophy and signal changes of the spinal cord are also a common and early finding (59) (Figure 5).

Molecular confirmation is by the detection of heterozygous *LMNB1* gene duplication or rarely deletion upstream of the *LMNB1* promoter that results in the overexpression of LMNB1 protein and an abnormal nuclear envelope (60, 61). Next-generation sequencing does not reliably identify this specific molecular finding, and hence targeted analysis for duplications and deletion upstream of the *LMNB1* gene is indicated in the appropriate clinical and radiological setting. Genotype-phenotype correlation have been described in a small cohort with earlier age at onset, more prominent cognitive impairment, and lack of dysautonomia in individuals with upstream deletion when compared with those with duplication (60). No curative treatment is available currently.

### Gordon Holmes syndrome

**Figure 6:**
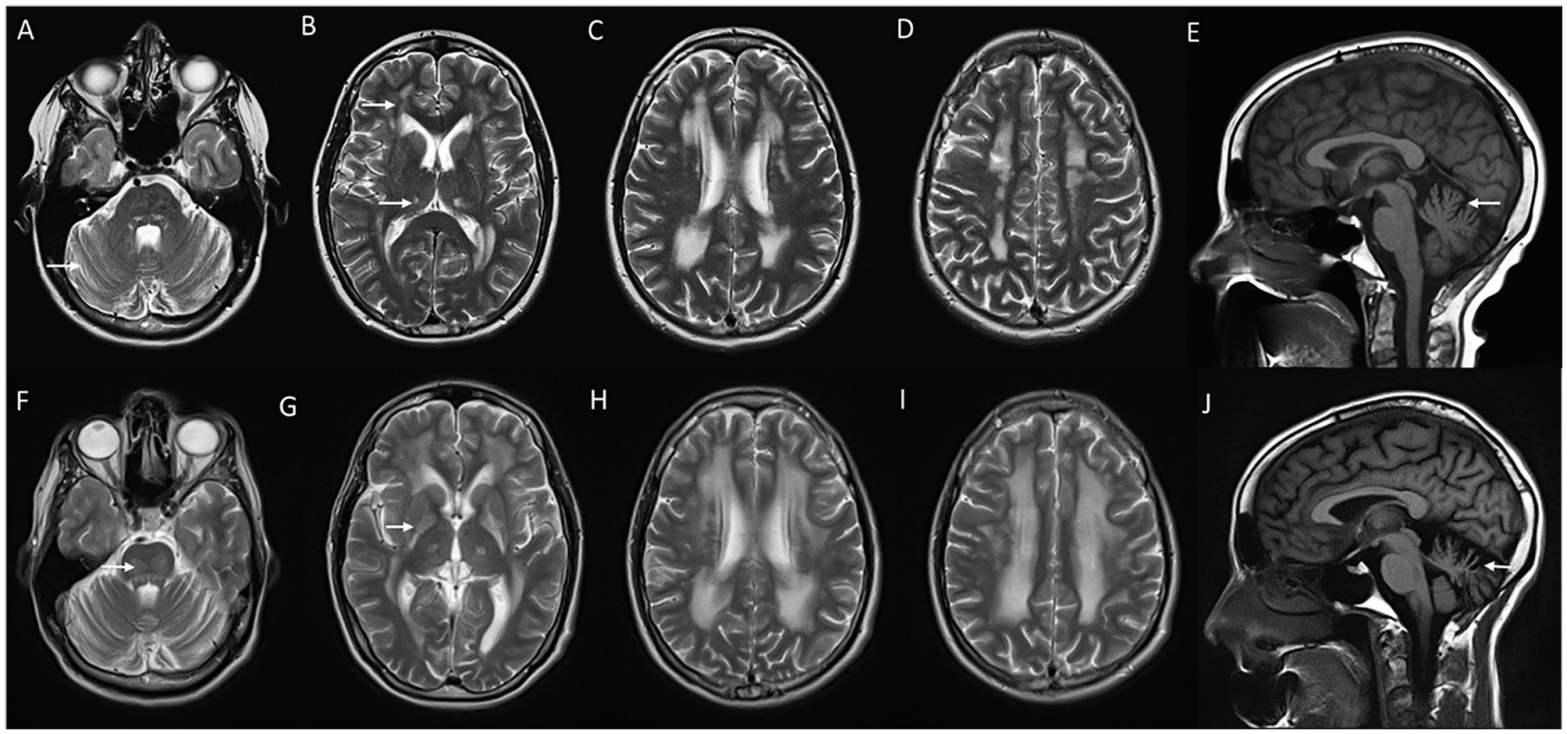
Serial neuroimaging findings in Gordon Holmes syndrome. MRI brain at age 31 **(A–E)** and 8 years later at age 39 **(F–J)** at corresponding sections shows the interval progression of findings. T2W axial images **(A–D, F–I)** show mild prominence of cerebellar foliae **(A)** with the further progression of cerebellar atrophy with the new appearance of brain stem changes (arrow in F), scattered frontal deep and periventricular white matter changes and thalamic involvement (arrows in B) with further progression in the extent of white matter changes along with the new appearance of globus pallidus and putaminal involvement [arrow in **(G)**], **(C, D, H, I)**. T1 sagittal images reveal interval progression of cerebellar atrophy [arrows in **(E, J)**].

Gordon Holmes syndrome (MIM# 212840) is a rare, distinct autosomal recessive leukodystrophy due to biallelic pathogenic variants in *RNF216* which code for E3 ubiquitin ligase (62). This was initially described as a syndromic association of cerebellar ataxia with hypogonadotropic hypogonadism (63). Endocrine manifestations usually occur in adolescence or early adulthood. Neurological manifestations are characterized by early adulthood onset progressive cerebellar ataxia with cognitive decline, chorea, behavioral issues, and spasticity (64). Brain MRI demonstrates progressive cerebellar and cerebral atrophy, with T2W hyperintense signal changes involving periventricular and subcortical white matter, globus pallidus, thalamus, and pons (Figure 6). Treatment is supportive and involves hormonal replacement (65, 66).

### *CSF1R*-related leukoencephalopathy

*CSF1R*-related leukoencephalopathy (adult-onset leukoencephalopathy with axonal spheroids and pigmented glia, MIM # 221820) is a primary microgliopathy with autosomal-dominant inheritance, caused by a heterozygous pathogenic variant in *CSF1R* (67). The age at onset of symptoms is usually in the early 40s, and the clinical course is rapid once the symptoms appear, with a reported mean disease duration of 6.8 years (68). The most common early symptoms are neuropsychiatric manifestations resembling behavioral variant fronto-temporal dementia, such as depression, anxiety, apathy, irritability, personality changes, and cognitive decline (67). This is followed by motor symptoms including progressive rigidity, bradykinesia, tremors, spasticity, ataxia, and seizures. Clinical features can rarely be predominated by motor symptoms without much behavioral or personality changes (67). Women tend to have an earlier age at the onset of clinical symptoms; however, there is no difference in the disease duration between women and men. Women with the earlier onset of symptoms usually have motor predominant clinical manifestations which are often misdiagnosed as multiple sclerosis in view of the overlap in clinical and imaging features (68). Clinical diagnostic criteria for early diagnosis and targeted genetic testing have been proposed (69).

**Figure 7:**
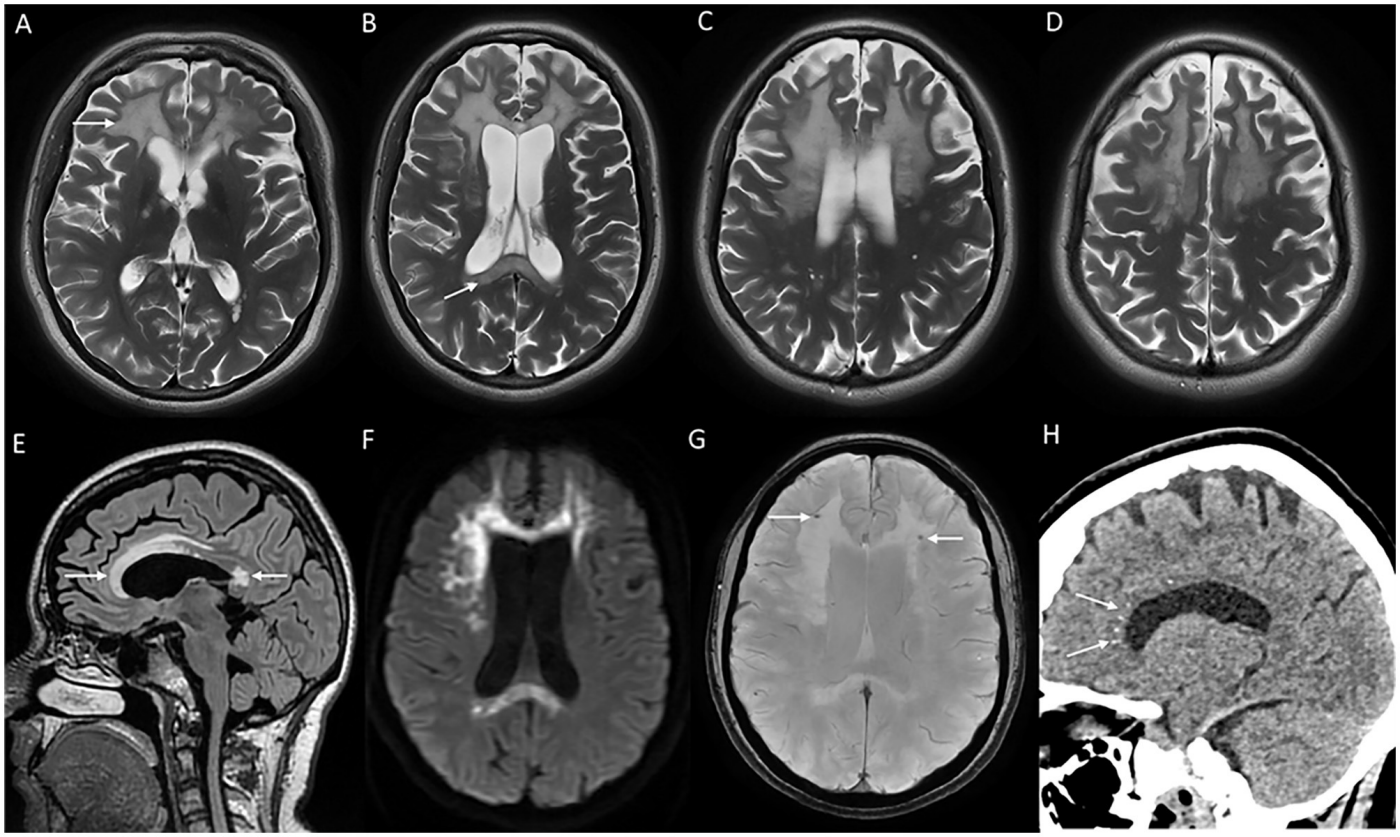
MRI brain findings of a patient with *CSF1R*-related leukoencephalopathy with T2W axial images **(A–D)** reveal confluent near symmetric bifrontal periventricular and deep white matter signal changes including the splenium of the corpus callosum [arrow in **(B)**], T2 FLAIR sagittal images **(E)** with signal changes involving rostrum, genu, anterior part of the body, and splenium of the corpus callosum; diffusion-weighted images **(F)** with heterogeneous high signal in frontal white matter and splenium; SWI **(G)** with microcalcifications in the frontal subcortical white matter, and CT coronal image **(H)** demonstrating stepping stone pattern of calcification involving the anterior part of the corpus callosum.

MRI brain reveals white matter changes in subcortical, deep, and periventricular regions in an asymmetric distribution, in addition to signal changes and thinning of the corpus callosum, and cerebral atrophy with the prominence of lateral ventricles and convexity subarachnoid spaces that is more prominent over frontal and parietal regions (Figure 7). Signal changes along the corticospinal tract in the posterior limb of the internal capsule and brain stem are commonly observed. The involved white matter shows a high signal in DWI sequences that probably indicates intramyelinic edema (70). Contrast enhancement is not observed, and the posterior fossa is usually spared. MRI scoring system has been established to assess the severity and longitudinal followup (71). CT brain and high-resolution gradient echo sequences demonstrate scattered punctate calcifications of the involved white matter adjacent to the frontal horn of lateral ventricles and in the parietal subcortical regions. A characteristic finding in the sagittal view is the pattern of calcification in the pericallosal white matter that has been described as a steppingstone appearance (70).

Phenotype–genotype correlation has not been established; however, seizures were noted more commonly in individuals with pathogenic variants in the proximal kinase domain of CSF1R (67). Hematopoietic stem cell transplantation has been attempted with the stabilization of motor, cognitive, and imaging scores (72).

### Neuronal intranuclear inclusion disease

Neuronal intranuclear inclusion disease (MIM # 603472) is an autosomal-dominant non-vascular leukoencephalopathy manifesting in adulthood, and occurs due to heterozygous pathogenic trinucleotide GGC repeat expansions in the 5′ untranslated region of *NOTCH2NLC* gene (13). The onset of symptoms ranges from the second to the eighth decade. Clinical features are characterized by cognitive decline, behavioral issues, recurrent encephalitis-like episodes, parkinsonism, tremors, muscle weakness, neuropathy, seizures, ataxia, and autonomic dysfunction (73). Common autonomic symptoms described are miosis, bladder dysfunction, vomiting, diarrhea, constipation, and orthostatic hypotension. Oculopharyngodistal myopathy and amyotrophic lateral sclerosis phenotypes have been described rarely (73). The disorder is broadly classified into dementia-dominant and muscle weakness-dominant phenotypes based on the earliest and prominent initial symptoms. Dementia presentation appears to be common in late-onset sporadic cases, and familial cases with symptom onset beyond 40 years of age, whereas muscle weakness is the prominent symptom in familial cases with symptom onset earlier than 40 years of age (73). Peripheral neuropathy and muscle involvement with elevated creatine kinase levels are noted in a significant proportion of affected individuals. In the muscle predominant group, symptoms of brain involvement can be delayed as long as two decades (13). Acute encephalitic-like episodes can mimic MELAS and present with focal neurological deficits and altered consciousness (73). Acute clinical and radiological findings in such presentations can be reversible. Longer repeats are associated with an earlier age of symptom onset (73).

**Figure 8:**
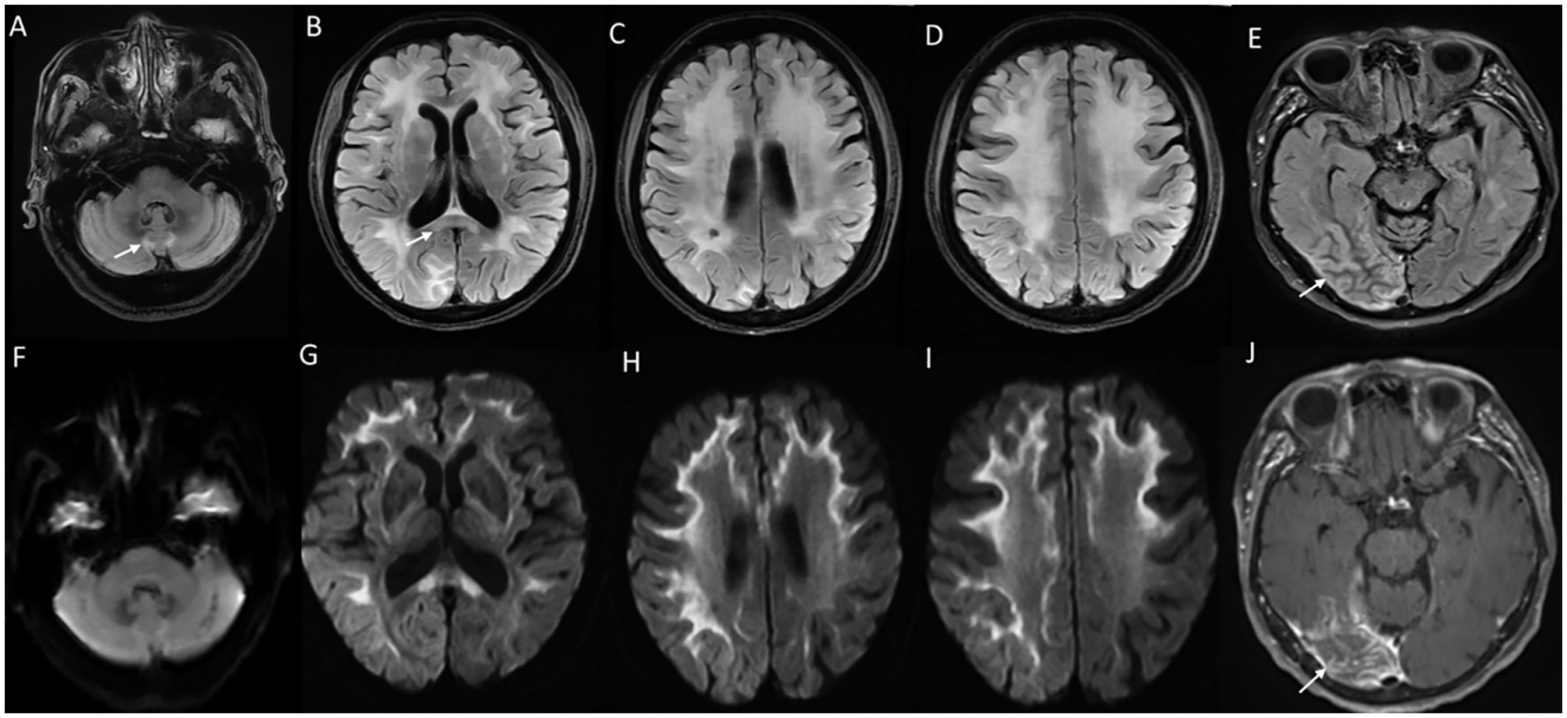
MRI brain findings in neuronal intranuclear inclusion disease (NIID). T2FLAIR sequences **(A–D)** reveal diffuse signal changes subcortical, periventricular, and deep white matter including splenium [arrow in **(B)**] and para-vermian region **(A)**, along with corresponding DWI sequences **(F–I)** with high signal in a curvilinear pattern at the cortical gray–white matter junction of the supratentorial region. Imaging of a patient with NIID during recurrent encephalitis-like presentation revealing swelling, and signal changes involving right occipital gray matter in the T2FLAIR images **(E)** and abnormal leptomeningeal enhancement in the post-contrast sequence **(J)**.

MRI brain findings are characterized by symmetric white matter hyperintensities in subcortical, deep, and periventricular white matter in frontal and parietal regions along with volume loss and ventricular dilatation (13, 73). Involvement of the external capsule, splenium, para-vermian region, and middle cerebellar peduncle is commonly seen (Figure 8). Involvement of these structures along with curvilinear DWI hyperintense lesions along the corticomedullary junction is probably a reliable imaging marker for the diagnosis of NIID (73). In the advanced stages, the corticomedullary DWI high-intensity signal appears to extend along the corticomedullary junction, but not into deep white matter (Figure 8). A small proportion of cases might lack diffusion changes. In acute encephalitic-like presentations, focal cortical hyperintensity and edema corresponding to the region of deficit are seen (13).

Accumulation of eosinophilic, hyaline, ubiquitin, and P-62 positive intranuclear inclusions in central and peripheral nervous systems and other organs including the skin is helpful in the pathological diagnosis (73). Clinical, radiological, and skin biopsy findings can significantly overlap with FXTAS, and hence the exclusion of FXTAS by molecular testing is essential (74). Acute encephalitic-like illness needs to be differentiated from viral infections and might show clinical and imaging response to steroids in the short term, though executive dysfunction and other features tend to progress slowly (73).

### Fragile X-associated tremor/ataxia syndrome

Fragile X-associated tremor ataxia syndrome (MIM # 300623) is a X-linked hemizygous adult-onset neurodegenerative disorder caused by CGG trinucleotide repeat expansion in the premutation range (55–200 repeats) in the 5′ UTR of the *FMR1* gene (75). While full expansion (>200 repeats) leads to methylation-coupled silencing of the gene and results in fragile X syndrome, expansions in the permutation range lead to increased mRNA production and toxicity resulting in intranuclear inclusions in neurons and astrocytes (75). This accumulation causes FXTAS and premature ovarian insufficiency before 40 years of age. Penetrance for FXTAS is not complete, and usually occurs in ~40% of men and 20% of women who carry the premutation allele (75). These individuals are at risk for having children with fragile X syndrome as the trinucleotide repeats could expand to the full mutation range in the next generation. In fact, many symptomatic individuals are diagnosed after a family history of the fragile X syndrome in their children or grandchildren. The absence of family history does not however rule out FXTAS (76).

Symptoms start from the fifth to sixth decade of life, and are characterized by progressive cerebellar ataxia, tremors, cognitive decline, parkinsonism, neuropathy, behavioral issues, and autonomic dysfunction (75). Varying combinations of postural, action, and intention tremors are usually seen. Cognitive involvement is characterized by frontal subcortical dementia with predominant executive deficits. Psychiatric comorbidities such as anxiety, depression, and obsessive–compulsive disorder are common. Eye movement abnormalities and a progressive supranuclear palsy-like phenotype have been described (75). Women with FXTAS tend to have prominent parkinsonism and less frequent tremors when compared to men (76).

**Figure 9:**
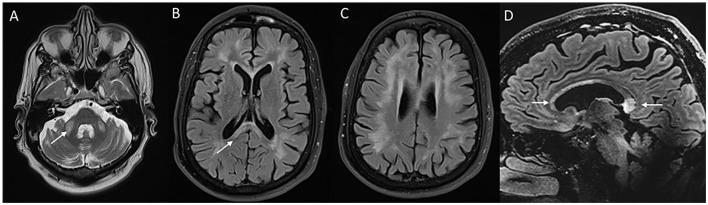
Neuroimaging in fragile X-associated tremor/ataxia syndrome. MRI T2W axial **(A)** reveals symmetric ovoid lesions in the middle cerebellar peduncle, along with prominent foliae suggestive of diffuse cerebellar atrophy. T2 FLAIR axial sections **(B, C)** show signal changes in the subcortical and white matter and splenium [arrow in **(B)**]. FLAIR sagittal section **(D)** shows anterior callosal and splenial hyperintensity.

MRI brain (Figure 9) reveals generalized cerebral and cerebellar atrophy along with symmetric T2W hyperintensities in the middle cerebellar peduncle, splenium of the corpus callosum, subcortical and periventricular white matter, brain stem, and deep cerebellar white matter (76). Diagnostic criteria have been established with the combination of clinical and imaging features (76, 77). MRI brain findings in a small proportion of affected individuals especially women may lack MCP signal changes. Clinical and radiological features can mimic NIID, as discussed above. Treatment is essentially supportive.

### Cerebrotendinous xanthomatosis

Cerebrotendinous xanthomatosis (MIM# 213700) is an autosomal recessive disorder of bile acid and cholesterol metabolism caused by biallelic pathogenic variants in *CYP27A1* that codes for the mitochondrial enzyme sterol 27-hydroxylase (78). Clinical manifestations are characterized by a spectrum of neurological and systemic manifestations. Systemic manifestations predominate early in the disease course with manifestations often starting from childhood with chronic diarrhea, cholestatic jaundice, and cataract. Tendon xanthomas develop in the second to third decade of life. Neurological manifestations usually appear by adolescence to early adulthood with progressive cerebellar ataxia, cognitive decline, epilepsy, spasticity, dystonia, palatal myoclonus, parkinsonism, and neuropsychiatric features (79). A rare spinal presentation called spinal xanthomatosis has been described with slowly progressive myelopathy with corticospinal and long-tract involvement (80). Other systemic manifestations include premature atherosclerosis, osteoporosis, and respiratory insufficiency (78).

**Figure 10:**
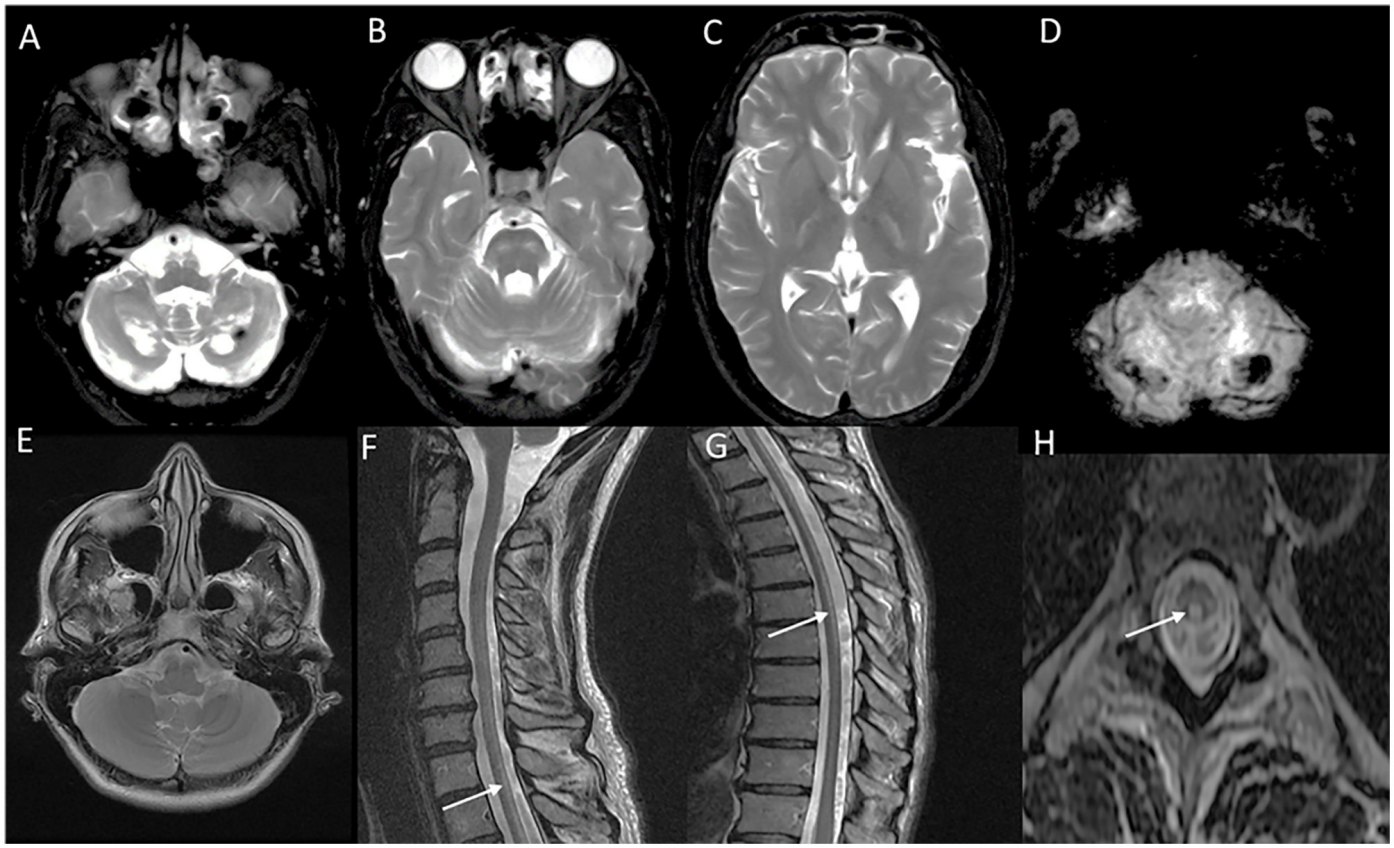
Neuroimaging finding in cerebrotendinous xanthomatosis **(A–D)** and spinal xanthomatosis **(E–H)**. T2 axial images **(A–C)** in a patient with progressive cerebellar ataxia, cognitive decline, malabsorption, and cataract related to CTX show heterogeneous signal changes in the dentate nucleus and surrounding cerebellar white matter **(A)**, and symmetric signal changes involving corticospinal tract in the brain stem **(B)** and posterior limb of the internal capsule. SWI images **(D)** show blooming in the cerebellar white matter suggestive of lipid deposition. Imaging **(E–H)** of an individual with progressive spastic paraparesis due to the spinal variant of CTX (spinal xanthomatosis): Normal brain imaging including posterior fossa **(E)**. T2 sagittal of spine revealing long segment linear hyperintensity along the posterior cervical **(F)** and thoracic cord **(G)**. Corresponding T2 axial section with signal changes in the dorsal column **(H)**.

Brain imaging reveals symmetric hyperintensity involving the dentate nucleus, corticospinal tracts in pons, and cerebral peduncles, and signal changes in cerebral and deep cerebellar white matter, along diffuse cerebral and cerebellar atrophy (Figure 10). Variable T2 hypointensity and blooming in susceptibility-weighted images in the dentate nucleus are also seen (79). MRI in spinal xanthomatosis usually reveal long segment signal changes along the spinal cord restricted to lateral and dorsal columns (80).

The elevated level of plasma cholestenol is the characteristic metabolic findings. Other lab findings include normal to reduced cholesterol levels, and increased levels of bile alcohols in urine (78). Long-term treatment with chenodeoxycholic acid is effective in reversing the manifestations in most cases. Early identification and timely initiation of treatment help with complete recovery (79).

### Hypomyelinating leukodystrophies

Hypomyelinating leukodystrophies are clinically and genetically heterogeneous disorders with the common pathology being permanent deficits in CNS myelin formation that result in eventual reduced myelin content (81). Symptoms manifest across all age groups, and adolescence and adult-onset symptoms are not uncommon. Common clinical presentation in late-onset hypomyelinating leukodystrophies is a combination of ataxia and spasticity, with most affected individuals having stable symptoms for decades before experiencing a slow progression of symptoms (81). Episodic worsening with inter-current illness, and improvement to baseline neurological status is known. Cognition is relatively preserved when compared with the extent of motor disability (81). A range of systemic associations such as hypodontia and endocrine abnormalities are known with hypomyelinating leukodystrophies. Advances in molecular testing have broadened the genetic landscape of hypomyelinating leukodystrophies with several implicated genes that code for structural myelin protein, cytoskeletal proteins, and other genes involved in transcription and translation (82). A few disorders that are encountered in adult practice are Pelizaeus-Merzbacher disease (PMD), Pelizaeus-Merzbacher-like disease (PMLD), and *POLR3*-related disorders (82).

Neuroimaging clinches the diagnosis and is often the initial clue for hypomyelinating leukodystrophies as the clinical symptoms are non-specific and overlap with several other static and degenerative neurologic disorders. Hypomyelination in imaging is characterized by limited myelin content that is depicted as mild T2 hyperintensity and variable (usually iso, hyper, or mild hypo) intensity in T1W images, in contrast to demyelinating disorders that display marked T2 hyperintensity and corresponding T1 hypointensity (81). Apart from the diagnosis of hypomyelination, imaging can provide valuable diagnostic clues based on the regions involved and additional features (81). No definitive treatment options are available at this time, though there are anecdotal reports of stem cell transplants in the early onset and severe cases.

#### PMD and PMLD

**Figure 11:**
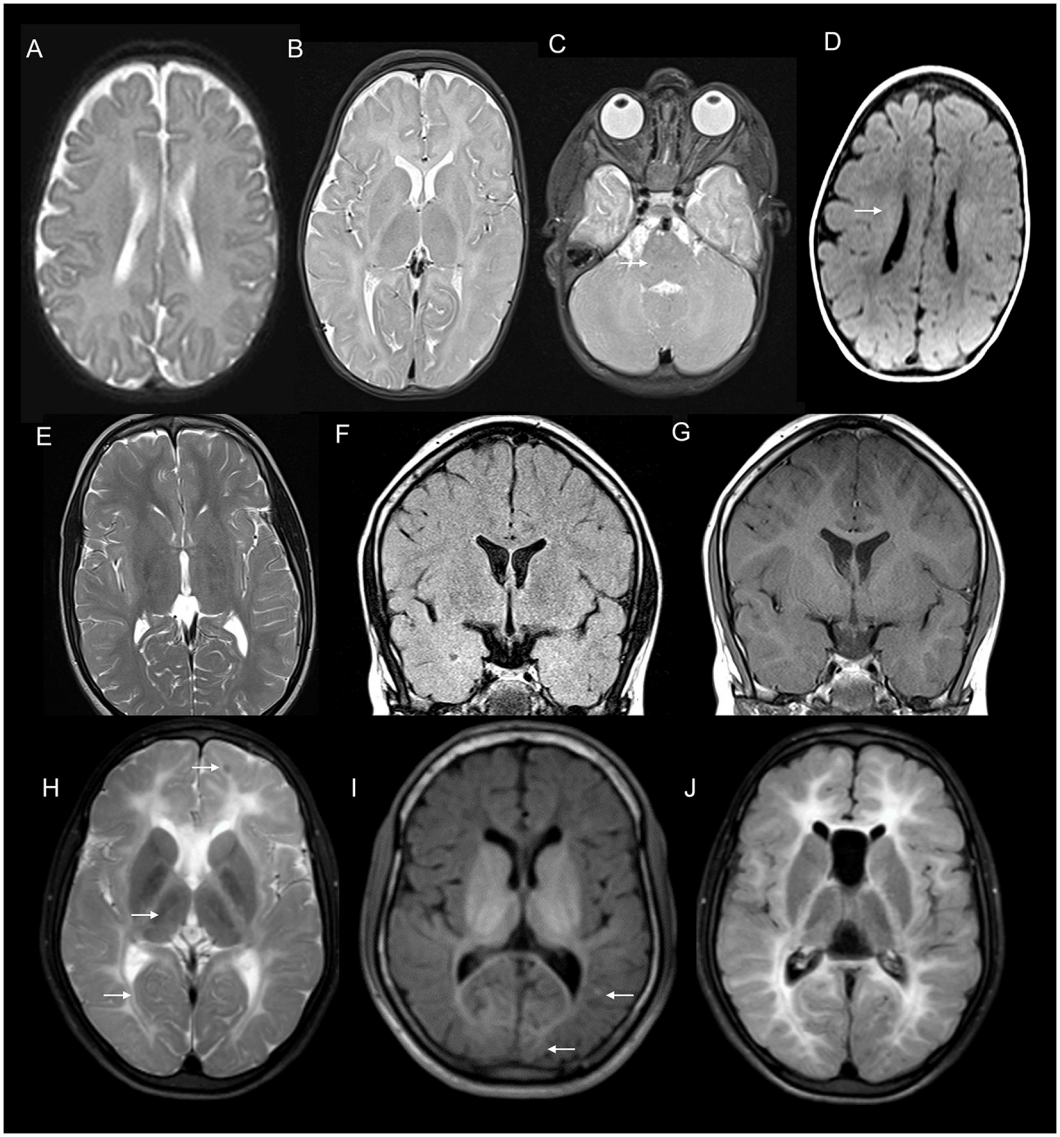
MRI features of hypomyelinating leukodystrophies. MRI features of an adolescent with spastic ataxic syndrome due to PMLD with T2 axial at 1 year **(A)** and 7 years **(B, C)** show near complete absence of myelination on the initial scan with no progression on the second scan. Note the diffuse pontine involvement in image C which is more common in PMLD than PMD.T1 axial image **(D)** shows hypo intensity of the white matter except in centrum semiovale. 18 q-syndrome in a 15 year old; T2 axial **(E)** shows reduced myelination than expected for age with a washed out appearance on FLAIR **(F)** but the preservation of hyperintensity on T1 images **(G)**. Combination of these signal abnormalities on various sequences is highly suggestive of hypomyelination. MRI brain findings of an individual with *POLR3*-related hypomyelination with T2 **(H)**, T1 **(I)**, and FLAIR **(J)** axial images showing diffuse hypomyelination with sparing optic radiations and patchy myelin islands. T2 also shows hypo intensity of ventrolateral thalamus due to the relative preservation of myelin.

PMD (MIM# 312080) is the prototype hypomyelinating disorder due to the duplication of the *PLP1* gene with X-linked inheritance. Clinical manifestations of PMD range from early severe connatal forms to adult-onset disorders with slow neurologic decline (81). MRI usually shows diffuse hypomyelination (Figure 11). PMLD (MIM# 608804) is a clinically and genetically heterogeneous disorder with symptoms similar to, but less severe than PMD (81). Pontine and dentate hilar hyperintensity in imaging, in addition to the diffuse cerebral and cerebellar hypomyelination differentiates PMLD from PMD (Figure 11). Several genes are implicated with the most common one being *GJC2* (81) with autosomal recessive inheritance.

#### *POLR3*-related disorders

*POLR3*-related hypomyelinating disorders are caused by defects in the RNA polymerase complex due to biallelic pathogenic variants in *POL3RA, POL3RB*, or *POLRC* (81) (MIM # 607694, 614381, 616494). Systemic associations such as hypodontia, oligodontia, hypogonadotropic hypogonadism, and severe myopia are seen to a variable extent in this group of disorder (81). Neuroimaging shows relatively better myelination of specific structures such as ventrolateral thalamus, optic radiation, and pyramidal tracts in the posterior limb of the internal capsule when compared with other regions of the brain (Figure 11). Cerebellar atrophy is more pronounced than the other group of hypomyelinating leukodystrophies (81).

### Genetic vasculopathies

Hereditary cerebral small vessel diseases (CSVD) are a heterogeneous group of disorders that result in progressive white matter changes in imaging, along with multiple lacunar infarcts and microhemorrhages (83). Though the initial manifestation tends to be recurrent transient ischemic attacks or stroke with a step ladder pattern of neurologic decline, clinical features eventually progress to involve cognitive decline, behavioral issues, gait dysfunction, and bladder disturbances (83). Common genetic vascular leukoencephalopathies are cerebral autosomal-dominant arteriopathy with subcortical infarcts and leukoencephalopathy (CADASIL), cerebral autosomal recessive arteriopathy with subcortical infarcts and leukoencephalopathy (CARASIL), *COL4A1*-related CSVD, cathepsin A-related arteriopathy with strokes and leukoencephalopathy (CARASAL), *SNORD118*-related leukoencephalopathy with cysts and calcifications (LCC), *FOXC1/FOXF2*-related disorders*, CTC1*-related cerebroretinal microangiopathy with calcifications and cysts, *TREX1*-related vasculopathy, retinal, with cerebral leukoencephalopathy and systemic manifestations, methylenetetrahydrofolate reductase deficiency, homocystinuria, and Fabry disease (84). Clinical manifestations are heterogenous with symptom onset usually from the third to fourth decade in most disorders. Diagnosis is challenging, as the clinical and imaging findings significantly overlap with sporadic CSVD due to vascular risk factors such as hypertension, hyperlipidemia, and diabetes mellitus which are quite common in the adult population (83).

Common imaging findings are progressive cerebral white matter lesions, multiple lacunes, scattered cerebral microbleeds, dilated perivascular spaces, and cerebral atrophy. Specific imaging findings and systemic associations can help in distinguishing these disorders (85). Though there are no curative treatments available at this time, strict control of modifiable vascular risk factors and usage of antiplatelet therapies when indicated help in slowing down the disease progression to some extent (83).

#### Cerebral autosomal-dominant arteriopathy with subcortical infarcts and leukoencephalopathy

**Figure 12:**
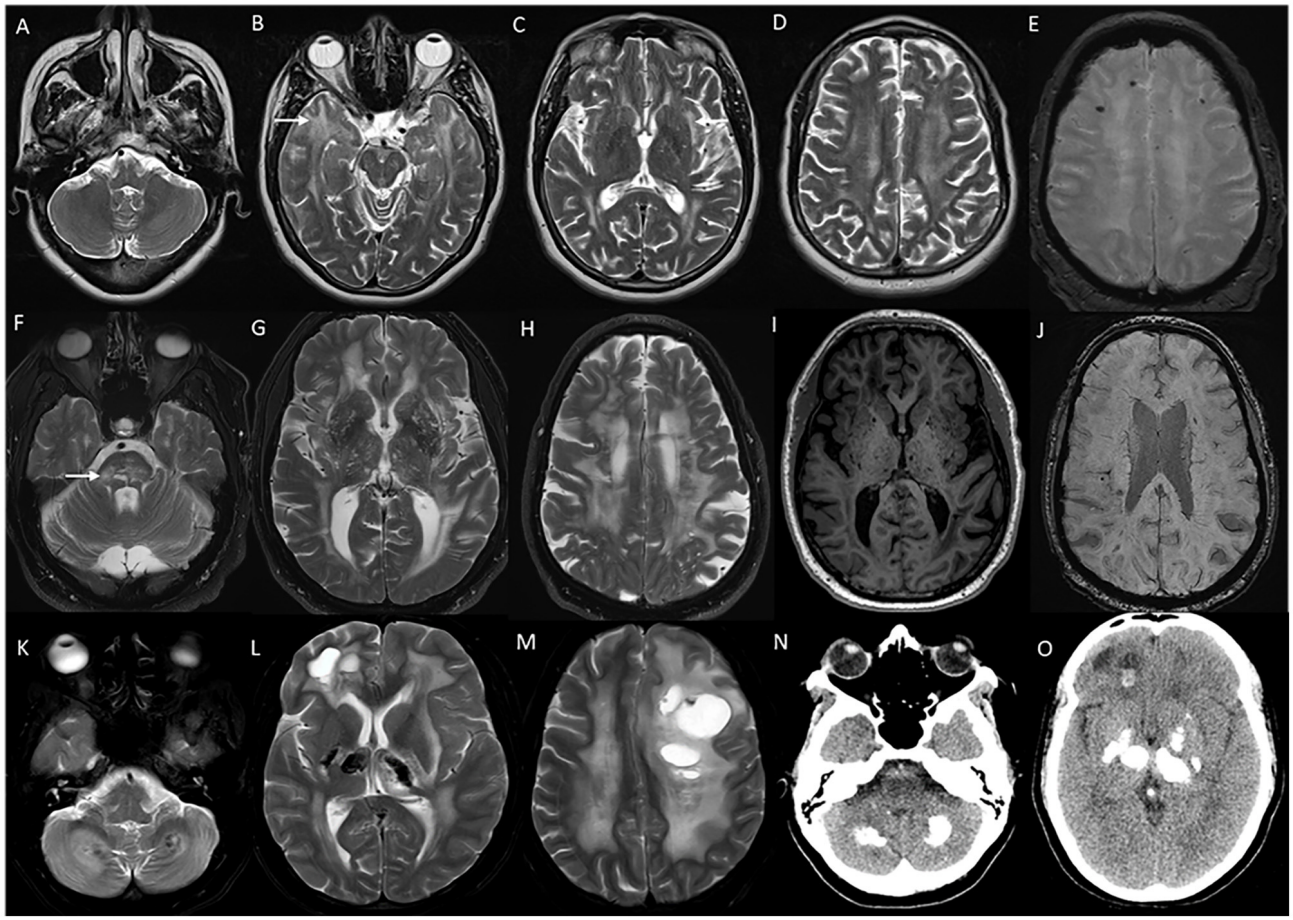
Imaging in genetic vasculopathies. MRI **(A–E)** in a patient with CADASIL demonstrating anterior temporal white matter [arrow in **(B)**], external capsule [arrow in **(C)**], and diffuse periventricular and subcortical white matter involvement in high frontoparietal regions, along with multiple foci of microhemorrhages in SWI sequences **(E)**. MRI brain **(F–J)** axial images in a patient with pontine autosomal-dominant microangiopathy with leukoencephalopathy (PADMAL) due to pathogenic variant in *COL4A1* demonstrating confluent white matter changes in supratentorial region, and pons [arrow in **(F)**], along with scattered lacunar infarcts, prominent vascular spaces **(I)** and microhemorrhages **(J)**. MRI findings in an individual **with** leukoencephalopathy, calcifications, and cysts: T2W axial images **(K–M)** with heterogeneous white matter changes in the cerebellum, supratentorial white matter, and thalamus along with cystic lesions of various sizes. CT sections **(N, O)** with calcifications in corresponding regions.

CADASIL (MIM# 125310) is probably the most common monogenic CSVD with autosomal-dominant inheritance, and is caused by a heterozygous pathogenic variant in the *NOTCH3* gene (84). Clinical manifestations are early-onset recurrent migraine episodes with prolonged visual, sensory, or motor auras, frequently associated with hemiplegic episodes starting in the second decade, followed by strokes and transient ischemic attacks from the fourth decade. These symptoms are eventually followed by progressive subcortical dementia, gait impairment, bladder, and bowel dysfunction by around sixth decade of life (84). Psychiatric manifestations such as mood disorders, and seizures are common (85). Imaging features are diffuse periventricular and subcortical white matter signal changes, multiple lacunar infarcts, and scattered microbleeds (Figure 12). White matter changes involving anterior temporal lobes and external capsules are considered characteristic of CADASIL, which helps to differentiate from acquired CSVD due to vascular risk factors (85). Skin biopsy reveals granular osmiophilic material in the walls of affected arterioles in the skin, and the molecular analysis confirms the diagnosis (84).

#### Cerebral autosomal recessive arteriopathy with subcortical infarcts and leukoencephalopathy

CARASIL (MIM # 600142) is caused by biallelic pathogenic mutations in the *HTRA1* that leads to defective repression of transforming growth factor-β signaling (83). As the name implies, the inheritance pattern is autosomal recessive. Neurologic manifestations are similar to CADASIL except that the onset of strokes is much earlier, and migraine episodes are less frequent in CARASIL (85). Recurrent strokes appear in the second decade, and dementia appears between the ages of 30 and 40. Systemic associations are alopecia and low back pain due to spondylolysis deformans. Alopecia is noticed from adolescence, and low back pain starts from the second to third decade (84). Cranial MRI findings are similar to that in CADASIL patients. Unlike CADASIL, a skin biopsy is not informative. Recently, heterozygous carriers of specific *HTRA1* mutations have been found to have CSVD manifestations at a later age and lesser severity when compared to individuals with autosomal recessive inheritance (83).

#### *COL4A1/2*-related CSVD

*COL4A1/2*-related CSVD (MIM # 175780, 614483) is caused by heterozygous pathogenic variants in *COL4A1 or COL4A2*, and is inherited in an autosomal-dominant manner (84). A spectrum of neurologic manifestations ranging from porencephaly and infantile hemiparesis to adult-onset CSVD and intracranial bleeds is known (84). The mean age of onset of stroke in adult-onset presentation is 36 years. Migraine and asymptomatic intracranial aneurysms are common (86). Systemic associations are known with the involvement of eyes (cataracts, Axenfeld–Rieger anomaly, and retinal vascular tortuosity), kidney (hematuria and cysts), and muscle cramps (86). Brain imaging findings are diffuse periventricular and subcortical white matter changes along with brain stem signal changes, with sparing of the temporal and occipital lobes (Figure 12). Microbleeds are seen in both deep white matter and lobar locations (86). Pontine autosomal-dominant microangiopathy and leukoencephalopathy (PADMAL) (MIM # 618564) is a specific clinico-radiological entity that results from mutations in the 3′ UTR of *COL4A1*, and presents with additional clinical findings of ataxic hemiparesis, and imaging finding of prominent pontine lacunes along with the cerebral hemispheric changes (86).

#### Leukoencephalopathy, calcifications, and cysts

LCC or Labrune syndrome (MIM # 614561) is characterized by the neuroimaging triad of leukoencephalopathy, parenchymal calcifications, and cysts (87). LCC occurs as a result of cerebral microangiopathy with autosomal recessive inheritance due to biallelic pathogenic variants in the *SNORD118* gene (87). The age at which symptoms appear varies greatly, and symptom onset as late as age 70 has been described, though most affected individuals manifest with some symptoms by adolescence or early adulthood. Clinical manifestations are seizures, progressive pyramidal, extrapyramidal, cerebellar signs, increased intracranial pressure, ischemic, and hemorrhagic strokes (87, 88). Acute or subacute worsening due to hemorrhage and mass effect due to enlargement of cysts can occur rarely, warranting surgical intervention (88).

Typical neuroimaging findings are asymmetric scattered cysts of varying shapes and sizes, calcifications, and white matter changes in subcortical white matter, basal ganglia, thalamus, and cerebellum (Figure 12). Careful review of gradient echo sequences, or a CT scan is warranted to identify the calcifications (87). Astrocytomas and parasitic infections such as neurocysticercosis are the differentials to be considered in the appropriate clinical scenario. Radiological features are indistinguishable from *CTC1*-related cerebroretinal microangiopathy with calcifications and cysts (CRMCCs) or Coats plus syndrome; however, CRMCCs usually have associated exudative vitreoretinopathy and systemic findings such as skeletal involvement, gastrointestinal bleeding, portal hypertension, and progeroid features (89).

Variable clinical and radiological therapeutic response to Bevacizumab has been described in the literature (90). Surgical decompression for progressive compressive symptoms due to enlarging cysts needs to be carefully considered on a case-to-case basis (87, 90).

### *CLCN2*-related leukoencephalopathy

*CLCN2*-related leukoencephalopathy (MIM# 615651), also known as leukoencephalopathy with ataxia (LKPAT) is inherited in an autosomal recessive manner and is caused by a defect in water and ion homeostasis in the brain, resulting in intramyelinic edema (91). Clinical features are cerebellar ataxia, spasticity, mild cognitive deficits, and recurrent headaches. Non-neurological manifestations involving eyes with chorioretinopathy and optic neuropathy are common (91). Hearing loss, tinnitus, vertigo, male infertility, and paroxysmal kinesogenic dyskinesia have also been described. Both pediatric and adult-onset symptoms are known, and the clinical course is usually indolent or slowly progressive (91).

MRI brain findings are characteristic, and show signal changes with restricted diffusion involving the posterior limb of the internal capsule and pyramidal tracts in the brain stem, along with the involvement of crus cerebri, central tegmental tract, decussation of the superior cerebellar peduncle, middle cerebellar peduncle, corpus callosum, dentate nucleus, and cerebellar white matter (91). No specific treatment options are available at this time.

### Adult polyglucosan body disease

Adult polyglucosan body disease (MIM# 615651) is an autosomal recessive adult-onset neurodegenerative disorder due to biallelic pathogenic variants in the *GBE1* gene (92). Symptoms occur in the fourth to fifth decade of life with prominent autonomic dysfunction in the form of neurogenic bladder and orthostatic hypotension, gait difficulties with both upper and lower motor neuron involvement, motor sensory axonal neuropathy, and mild executive dysfunction (92). Bladder symptoms are often the initial manifestation, and can antedate gait disturbances even by one to two decades (92). Atypical forms with relapsing-remitting clinical course, subacute presentations, and early-onset liver and neuromuscular disease have been reported (92).

MRI brain reveals hyperintense signal changes in the periventricular, deep, and subcortical supratentorial white matter, pyramidal tract, and medial lemniscus in the pons and medulla, along the cerebellar peduncles, external capsule, and dentate nucleus, as well as a progressive diffuse cerebral, cerebellar, and spinal cord volume loss. Prominent medullary and cervical cord atrophy without any supratentorial white matter changes have been described (93).

Diagnosis is established by skin or sural nerve biopsy demonstrating periodic acid-Schiff positive and diastase-resistant material, and is confirmed by molecular studies that demonstrate biallelic pathogenic variants in *GBE1* *(**92**)*. There are no curative treatment options available at this time.

### Vanishing white matter disease

Vanishing white matter disease (MIM# # 603896, 620312, 620313, 620314, 620315) is an autosomal recessive leukoencephalopathy caused by pathogenic variants in one of the *EIF2B1-5* genes (94). Initial presentation in adulthood is not uncommon, with symptom onset described as late as 55 years of age (94). Clinical manifestations in adult-onset presentations are psychiatric symptoms, dementia, motor symptoms including ataxia and spasticity, seizures, and headaches (94). Similar to childhood presentations, varying periods of stability and episodic deterioration with encephalopathy precipitated by febrile infections, minor head trauma, and other stressors are known. Affected females show signs of premature ovarian failure (94).

Neuroimaging demonstrates abnormal T2 hyperintense signal changes involving all of the supratentorial cerebral white matter with progressive rarefaction and cystic degeneration, that typically appears markedly hypodense in FLAIR images similar to that of CSF intensity (94). T1 and FLAIR images show radiating stripes within the areas of rarefaction that represent remaining tissue strands. Variable cerebellar atrophy is seen in late stages (94). Treatment is limited to supportive management and avoidance of trauma and febrile infections.

### Mitochondrial leukoencephalopathies

Neurological manifestations including leukoencephalopathy are one of the prominent features in many of the mitochondrial disorders that are caused by pathogenic variants in both the nuclear genes that encodes mitochondrial proteins, and the mitochondrial genome (95). Clinical manifestations range from early-onset severe leukoencephalopathies to late onset slowly progressive leukodystrophies, along with multisystemic manifestations. Several mitochondrial disorders can have initial symptom onset and presentation in adolescence and adulthood (95). Some of the common mitochondrial disorders encountered in adult neurologic practice are mitochondrial encephalomyopathy with lactic acidosis and stroke-like episodes (MELAS), Kearns–Sayre syndrome, Leigh disease, mitochondrial neurogastrointestinal encephalomyopathy (MNGIE), myoclonic epilepsy with ragged red fibers (MERRF), Leber hereditary optic neuropathy, pyruvate dehydrogenase complex deficiency, polymerase gamma (*POLG*)-related disorders, and various other mitochondrial DNA depletion syndromes (95).

Usual neuroimaging findings are white matter signal changes in supratentorial brain parenchyma sometimes associated with cystic changes and rarefaction, basal ganglia and thalamic signal changes, brain stem involvement, cerebellar white matter changes, and cytotoxic edema during stroke-like episodes (95). MR spectroscopy in the region of abnormal signal changes and sometimes even in the normal-appearing brain regions demonstrating elevated lactate provides additional support to the diagnosis of mitochondrial disorders (95). Although some of the specific clinical manifestations and a corroborative radiologic pattern pinpoint a specific mitochondrial disorder, the manifestations can at times be non-specific, and difficult to diagnose. There should be a high index of suspicion for mitochondrial disorders in the appropriate clinical and imaging context, as this has obvious therapeutic implications, with both use of mitochondrial megavitamins and avoiding some of the medications that are deleterious to mitochondrial function.

Mitochondria rely on several of the nuclear-encoded proteins for the transcriptional and translational regulation of their own housekeeping genes. Mutations in these nuclear-encoded mitochondrial genes, specifically the aminoacyl transfer RNA synthetases are increasingly being recognized as the cause of progressive neurologic disorders, and initial presentation in adulthood is known with this group of disorders. A detailed discussion of the mitochondrial disorders is beyond the scope of this study; however, we discuss two disorders with prominent leukoencephalopathy presentation in adulthood: leukoencephalopathy with brainstem and spinal cord involvement and lactate elevation due to *DARS2* variants, and *AARS2*-related leukodystrophy.

#### Leukoencephalopathy with brain stem and spinal cord involvement and lactate elevation

Leukoencephalopathy with brain stem and spinal cord involvement and lactate elevation (MIM# 611105) is caused by biallelic pathogenic variants in the *DARS2* gene, that encodes the mitochondrial aspartyl-transfer RNA synthetase (96). Inheritance is autosomal recessive. Clinical manifestations are characterized by insidious onset and gradually progressive cerebellar and sensory ataxia, and spasticity. Lower extremities are more prominently involved than the upper extremities. The age of onset of clinical symptoms is usually in late childhood or adolescence, though the onset of the motor deterioration in adulthood is not uncommon (96). Cognitive decline, epilepsy, and subacute neurologic decline with febrile illness, and head trauma are known (96).

Brain MRI findings are characteristic, and there is an established MRI-based criteria for the diagnosis of LBSL (96). Classic neuroimaging findings in the supratentorial region are the confluent hyperintense signal changes in the periventricular, deep white matter, and along the corticospinal tract in the posterior limb of the internal capsule extending down to the brain stem, along with signal changes in medial lemniscus and intraparenchymal trajectories of the trigeminal nerves, superior cerebellar peduncle, and cerebellar white matter (96). Spinal cord imaging reveals signal changes along the whole cord with the hyperintensities restricted to the lateral corticospinal tract and dorsal column. Sparing of the posterior fossa and brain stem has been noted especially in early-onset cases. MR spectroscopy demonstrates elevated lactate peak; however, the absence of lactate peak in areas of high signal intensities has been documented in the literature (96). Treatment is supportive, and includes the avoidance of head trauma and intercurrent infections.

#### *AARS2*-related leukoencephalopathy

*AARS2*-related leukoencephalopathy (MIM# 615889) is inherited in an autosomal recessive manner, and is caused by pathogenic variants in *AARS2* which codes for mitochondrial alanyl transfer RNA synthetase 2 (97). Onset of symptoms usually occurs by the second to fourth decade. Cognitive decline and behavioral manifestations are the initial symptoms that are followed by cerebellar ataxia, spasticity, and extrapyramidal features such as rigidity, tremors, and bradykinesia (97). Psychiatric manifestations such as depression, anxiety, and personality changes are common. Ovarian failure is common in affected females that led to the coining of the past terminology ‘ovario-leukodystrophy' for this disorder (97).

MRI brain findings are periventricular and deep white matter changes in frontoparietal regions with relative sparing of the central area and subcortical U fibers with some asymmetry (98). Involvement of corpus callosum and signal changes along the corticospinal tract are commonly seen. Diffusion-weighted images demonstrate discrete areas of increased signal changes in deep white matter, also called deep white matter diffusion dots (97, 98). Significant clinical, radiological, and histopathologic overlap exists with *CSF1R*-related leukoencephalopathy, though there are some subtle differences (98). Another phenotype known with *AARS2*-related mutation is dilated cardiomyopathy with both early and late-onset cases being described (97).

### Other rare leukodystrophies

Several other rare leukodystrophies that are encountered in adulthood and are not discussed separately in this article are L2-hydroxyglutaric aciduria due to pathogenic variants in *L2HGDH* with autosomal recessive inheritance (MIM# # 236792), juvenile or atypical Canavan disease due to biallelic pathogenic variants in *ASPA* with autosomal recessive inheritance (MIM# 271900), megalencephalic leukoencephalopathy with subcortical cysts due to pathogenic variants in *MLC1* with autosomal recessive inheritance (MIM# 604004), *AARS1*-related leukoencephalopathy due to pathogenic variants in *AARS1* with autosomal-dominant inheritance (MIM# 619661), and Sjogren–Larsson syndrome due to pathogenic changes in *ALDH3A2* with autosomal recessive inheritance (MIM# 270200).

Despite clinical and radiological overlaps in these disorders, there are several pointers in the prominent neurological symptoms and systemic syndromic associations that would help us to suspect a specific leukodystrophy. These are shown in Tables 2, 3 and would be helpful in daily practice. Broader details including the genes and biochemical testing are shown in Supplementary Table 1.

## Acquired mimics

Acquired mimics of genetic leukoencephalopathies often pose a diagnostic challenge. The age of presentation and temporal profile of symptom progression provide important diagnostic clues in addition to the relevant neuroimaging findings. Toxins (carbon monoxide, heroin, and cocaine) and drugs (metronidazole, chemotherapeutic agents such as fludarabine, and immunosuppressants such as cyclosporine) may cause structural alterations of white matter (99). These disorders may have a symmetric reduction in the ADC and restricted diffusion in the periventricular white matter. The restricted diffusion is attributable to intramyelinic edema and myelin vacuolation (100). Capillary endothelial injury could result in cytotoxicity and there could be an element of toxic demyelination. The initial extent of changes in the FLAIR sequences may be lesser in comparison to the DWI-ADC sequences (101). Early prompt recognition and discontinuation of the incriminating agent is paramount to improve outcomes.

Inflammatory and autoimmune causes of leukoencephalopathies need to be considered in cases with subacute onset and rapid progression. The diagnosis is often supported by the evidence of inflammation in cerebrospinal fluid (CSF), the presence of asymmetric MRI changes with patchy enhancement, and response to immunotherapy (11). Autoantibodies described include anti-myelin oligodendrocyte glycoprotein (MOG), anti-N-methyl-D-aspartate (NMDA), aquaporin 4 (AQP4), and anti-glial fibrillary acid protein (GFAP). A brain biopsy may be warranted in selected cases, and the histological features supporting inflammatory etiologies include the presence of lymphocytic infiltrates and microglial activation, a pattern consistent with non-demyelinating inflammation. Fibrinoid necrosis may be present in vasculitis. Histological features suggestive of other genetic leukoencephalopathies may be absent, and genetic tests are negative. An important fact to note is that patients with underlying genetic leukoencephalopathies may present with an additional acquired toxic/metabolic insult or an inflammatory disease.

Acute leukoencephalopathy with restricted diffusion can occur with infections (viral and bacterial meningoencephalitis) and hypoxic-ischemic brain injuries (102). Human immunodeficiency virus (HIV) and progressive multifocal leukoencephalopathy (PML) can present with imaging features showing diffuse leukoencephalopathy. Clinical features in both PML and HIV leukoencephalopathy include varying degrees of cognitive changes, visual impairment, weakness, ataxia, and/or seizures. The imaging features include multifocal, bilateral, asymmetrical lesions involving the subcortical and periventricular white matter, predominantly affecting the parietal-occipital lobes and involving the U fibers. CD8 encephalitis due to viral escape phenomena and autoreactive CD8 cell-associated inflammation is an increasingly recognized entity, and is characterized by the imaging findings of diffuse bilateral white matter signal changes which could be punctate or linear and perivascular and could be associated with post-contrast enhancement (103). A lumbar puncture and/or brain biopsy is often required to establish a definitive diagnosis and to optimize treatment in the setting of HIV-associated illness.

Hypoglycemia and posterior reversible encephalopathy syndrome (PRES) can present with changes mainly in the parieto-occipital regions (104). The changes in PRES are related to vasogenic edema, and are often reversible. Cytotoxic edema can occur due to vasospasm, endothelial injury, and tissue hypoxemia. Rarely, hemorrhagic changes can also occur. PRES can occur in an atypical pattern with the involvement of the brainstem, cerebellum, deep gray nuclei, and spinal cord. Causes of PRES apart from hypertension include autoimmune disease, immunosuppressive drugs (such as tacrolimus and cyclosporine), and organ transplantation (105). Chronic hepatic encephalopathies can have features of vasogenic edema in the white matter mainly involving the corticospinal tract (99).

**Figure 13:**
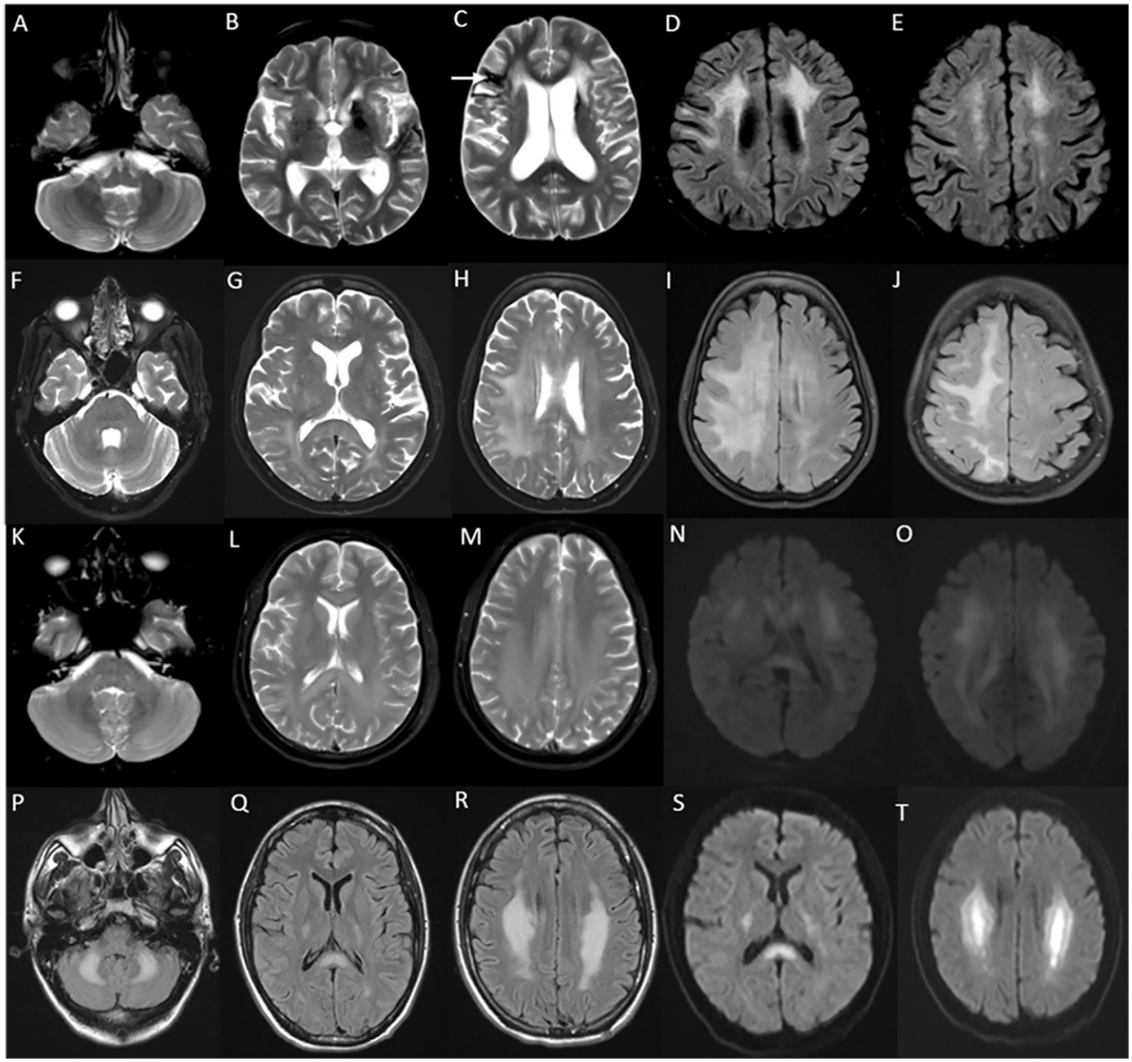
MRI axial images depicting acquired mimics for leukodystrophy. Images **(A–E)**in a patient with recurrent neurologic events due to neurosarcoidosis reveal confluent frontal deep and periventricular white matter changes and a focus of hemosiderin staining in the right frontal region [arrow in **(B)**]. Images **(F–J)** in a patient with 2 months history of neurologic decline revealing asymmetric right more than left diffuse white matter changes with the effacement of sulci in high frontoparietal regions with biopsy findings of Gliomatosis cerebri. Images **(K–O)** in a patient with acute encephalopathy following metronidazole administration for a gastrointestinal infection with imaging findings of faint T2W signal changes in the subcortical and periventricular regions and splenium with corresponding hyperintensity in diffusion images **(N, O)**, with prompt resolution of clinical and imaging findings, and diagnosis of metronidazole-related leukoencephalopathy. Images **(P–T)** in a patient with acute encephalopathy following the inhalation of heroin reveal signal changes in the dentate nucleus, subcortical, and deep white matter including splenium with restricted diffusion in corresponding DWI.

Gliomatosis cerebri can mimic demyelinating diseases, and is usually suspected when there is an expansion of involved structures on the MRI with minimal enhancement (106). MR perfusion and MR spectroscopy may be non-contributory, and a brain biopsy may be required for definitive diagnosis. Changes in DWI may give clues to the diagnosis of lymphomatosis cerebri (107). Unmyelinated white matter beyond 2 years of age is not an uncommon finding, and these terminal zones of myelination are an important mimic to be considered (108). Imaging findings in a few common acquired etiologies are shown in Figure 13. Given that these disorders are commonly encountered in day-to-day scenarios, it is prudent to consider these and exclude them in the appropriate clinical context before considering genetic etiologies.

## Treatment updates

Symptomatic and supportive treatment remains the mainstay of management in most adult-onset leukodystrophies. Pharmacotherapy for seizures, spasticity, sleep disturbances, and psychiatric comorbidities is essential. Physical, occupational and speech therapy, attention to general care including the optimization of nutritional needs and swallowing issues improve the quality of life. Periodic surveillance for endocrine involvement like adrenal involvement in X-ALD, and hypogonadism in *POLR3*-related disorders and Gordon Holmes syndrome are a vital part of systemic treatment in these disorders.

Recent advances in our understanding of the pathophysiology of various disorders had opened new treatment avenues such as hematopoietic stem cell transplantation, gene therapy, antisense oligonucleotides, enzyme replacement or enhancement therapy, and substrate reduction therapy. Hematopoietic stem cell transplantation in the early stages of adult-onset MLD and cerebral adrenoleukodystrophy is effective in arresting the clinical and radiological progression of the disease (21, 47). Though the indication for HSCT in these disorders is often extrapolated from childhood-onset presentations, the extent of clinical involvement, cognitive abilities, and radiological scoring provides guidance to select appropriate candidates. It should be noted that the clinical and radiological manifestations tend to progress until the engraftment is complete. Gene therapy has already been approved in various countries for MLD and X-ALD. Selecting ideal candidates for HSCT in juvenile and adult-onset Krabbe disease is difficult, as the clinical course of the disease is often unpredictable in late-onset presentations. Given the anecdotal evidence of beneficial effects, documentation of a progressive clinical course and elevated psychosine levels in plasma and CSF would help clinicians to individualize this treatment option in adult-onset Krabbe disease (27).

Clinical and radiological manifestations of CTX are reversible with long-term treatment with chenodeoxycholic acid in most patients when treatment is initiated early in the disease course. There is emerging evidence that HSCT can stabilize motor and cognitive abilities in individuals with *CSF1R*-related leukoencephalopathy (72). Strict control of modifiable vascular risk factors is helpful in slowing down the progression of inherited cerebral small vessel disorders. Individuals with certain leukodystrophies such as X-ALD, LBSL, ADLD, and VWMD tend to have a rapid downhill course with minor head trauma and intercurrent infections. Hence, the use of safety helmets, physical accommodations to prevent falls, and appropriate vaccinations to prevent febrile illnesses need to be considered in these disorders. Mitochondrial cocktail with megavitamins and antioxidants have been noted to be helpful in several mitochondrial disorders. HSCT, dialysis, and platelet transfusions are potential therapy options for MNGIE (109, 110). Clinical trials with gene therapy, substrate reduction therapies, and enzyme replacement therapies are ongoing for several leukodystrophies. Interested readers are requested to review the ongoing clinical trials for specific disorders at clinicaltrials.gov.

## Genetic testing

The diagnosis of leukodystrophy starts with a comprehensive personal and family history, neurological examination, and neuroradiologic review. In certain cases, with specific imaging, clinical and biochemistry findings, it may be possible to reach a definitive diagnosis prior to genetic testing. However, due to genetic heterogeneity, molecular testing is often required to confirm the diagnosis. Genetic testing can be targeted single gene testing followed by panel testing, or whole genome or exome testing including mitochondrial genome sequencing.

As genetic testing especially next-generation sequencing has become more widely available and affordable, the use of whole-exome (WES) and whole-genome sequencing (WGS) has become routine in the diagnosis of leukodystrophies. A study published in 2016 showed a yield of approximately 42% for the use of WES in a cohort of patients with unsolved white matter abnormalities (111). Performing whole-genome sequencing on the still unsolved cases increased the yield by an additional 11% (112). As gene discovery is happening rapidly, reanalysis of the raw data every 1–2 years can provide an additional yield of up to 10% (113).

It should be remembered, however, that for some disorders with copy number changes such as *LMNB1* and *PLP1*-related disorders, indels in *GBE1*, deep intronic variants, complex rearrangements, and repeat expansion disorders such as NIID, the routine NGS will not be sufficient. Awareness of the clinico-radiological spectrum of these disorders is important, as this needs targeted genetic testing with different methodologies. This encompasses a range of techniques such as multiplex ligation-dependent probe amplification (MLPA), gene-targeted microarray, capillary-array electrophoresis, quantitative PCR, long-range PCR, repeat-primed PCR, and southern blotting. Advances in molecular technologies have largely surpassed the need for invasive testing such as skin or brain biopsies. Regular reanalysis of the raw exome or genome data and periodic relook into the phenotype and evolution of imaging findings when embedded into the clinical practice would help to reduce the incidence of unsolved leukodystrophies in the long term.

## Challenges, opportunities, and future directions

Delay in the diagnosis of adult leukodystrophy is common, due to an overlap with acquired disorders such as infectious, post-infectious, inflammatory, autoimmune, toxic, and metabolic etiologies, and the high prevalence of confounding acquired risk factors in this population. The first step would typically be to rule out these acquired causes. Most often, the clinical scenario and course of the disease help in this step. Once a diagnosis is considered presumed genetic, the mode of inheritance when there is a positive family history can narrow the differential diagnosis. As discussed in the earlier part of this review, the absence of family history does not rule out inherited etiologies as reduced penetrance, misdiagnosis, and de novo gene variations are known with several adult leukodystrophies.

Awareness of the neuroradiologic pattern in the appropriate clinical context is paramount in deciding further diagnostic workup. Advanced imaging techniques like MR spectroscopy and Diffusion Tensor Imaging, and spine imaging supplement MRI Brain in further delineating the diagnosis. Artificial intelligence and machine learning in radiology are rapidly evolving, and would certainly play an important role in the diagnostic pathways in future. Radiological biomarkers and advanced imaging techniques would help in both diagnosis and monitoring response in treatment trials.

**Figure 14:**
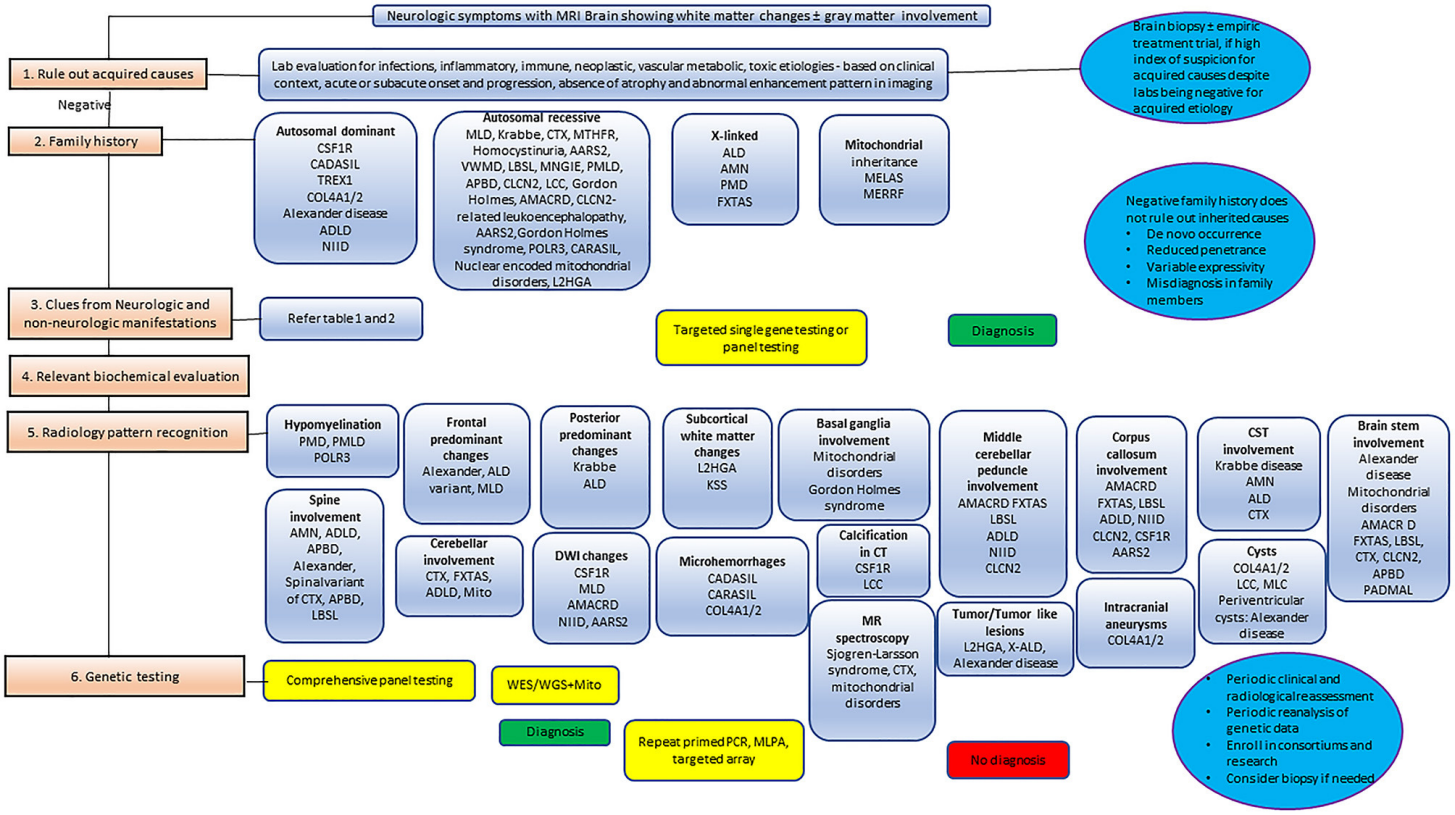
Algorithm for approach and diagnostic pathway of adult-onset leukodystrophies.

Focused biochemical analysis for diagnostic metabolite profile, substrate accumulation, and enzyme assays when available provides valuable guidance in choosing the genetic testing. Referral for genetic testing should be considered early in the course. The use of large next-generation panels or whole-exome or whole-genome testing should be offered early in the diagnostic odyssey of these patients. Figure 14 shows a proposed algorithm for approaching these disorders. Creating a bio-repository for undiagnosed adult-onset leukodystrophies, targeted and untargeted omics including RNA profiling, proteomics, metabolomics, and integration of these omics data to facilitate gene and biomarkers discovery is a felt need to advance this field.

A comprehensive team comprising of experts from neurogenetics, inherited metabolic disease, neuroinflammation, cognitive neurology, neuroradiology, and physical medicine and rehabilitation would be able to provide valuable inputs into the clinical management and research. This group of patients would be best served in such leukodystrophy clinic or center where the process from diagnosis to enrollment in natural history and potential clinical trials is streamlined. However, few such centers are available at this time. Patient advocacy groups, e.g., ADLD Center (https://adld.center/) and Krabbe connect (https://krabbeconnect.org/), have been important in bringing together clinicians, researchers, and pharmaceutical companies for specific leukodystrophies.

Treatment and management of this group of disorders remain a challenge. Few targeted treatments are available, and general approaches in management are usually followed. Rapid progress in our understanding of pathophysiology in several disorders has opened up new treatment avenues that are being explored in the pre-clinical trials. Precision and individualized medicines with ASOs and gene therapy are being investigated in various adult leukodystrophies, and the preliminary results are encouraging. We encourage families with leukodystrophies and the clinicians who care for them to periodically review ClinicalTrials.Gov for getting updates on trials that could be beneficial.

Consortiums focused on adult leukodystrophies and international collaboration can help foster research and clinical trial development for this unique and challenging group of disorders. The establishment of comprehensive adult leukodystrophy centers with a multidisciplinary approach would help in the evaluation and holistic management.

## Author contributions

KM contributed to the conceptualization of the article, write-up, and compilation of the images and overall review of the draft. AS, LD, SS, MT, SD, ZW, KW, RD, and RG contributed to the write-up and critical review of the manuscript. All authors contributed to the article and approved the submitted version.
