# Supplementary material for: Adult-onset leukodystrophies: a practical guide, recent treatment updates, and future directions
Source: Front Neurol. 2023 Jul 26;14:1219324. doi: 10.3389/fneur.2023.1219324 (PMC10410460; doi:10.3389/fneur.2023.1219324)
Supplement: Supplementary file 1 [file Table_1.DOCX]

Table 1: Summary of clinical, imaging and biochemistry features which are commonly associated with specific adult-onset leukodystrophies.

MLD, metachromatic leukodystrophy, X-ALD, X-linked adrenoleukodystrophy, AMN, adrenomyeloneuropathy, NIID, neuronal intranuclear inclusion disease, FXTAS, fragile X-associated tremor ataxia syndrome, CTX, cerebrotendinous xanthomatosis, PMD, Pelizaeus Merzbacher disease, PMLD Pelizaeus Merzbacher-like disease, CADASIL, cerebral autosomal dominant arteriopathy with subcortical infarcts and leukoencephalopathy, CARASIL, cerebral autosomal recessive arteriopathy with subcortical infarcts and leukoencephalopathy, LCC, leukoencephalopathy with cysts and calcifications, LBSL, leukoencephalopathy with brain stem and spinal cord involvement and lactate elevation. AR = autosomal recessive, AD = autosomal dominant, XR = X-linked recessive, XL = X-linked variable, VLCFA = very long chain fatty acids. Green boxes = common findings, blue boxes = anatomical regions which are particularly spared.
